# MyD88-dependent BCG immunotherapy reduces tumor and regulates tumor microenvironment in bladder cancer murine model

**DOI:** 10.1038/s41598-021-95157-6

**Published:** 2021-08-02

**Authors:** Nina M. G. P. de Queiroz, Fabio V. Marinho, Ana Carolina V. S. C. de Araujo, Julia S. Fahel, Sergio C. Oliveira

**Affiliations:** 1 Departamento de Bioquímica e Imunologia, Instituto de Ciências Biológicas, Universidade Federal de Minas Gerais, Belo Horizonte, MG Brazil; 2 Instituto Nacional de Ciência e Tecnologia em Doenças Tropicais (INCT-DT), CNPq MCT, Salvador, BA 31270-901 Brazil

**Keywords:** Tumour immunology, Applied microbiology

## Abstract

Bacillus Calmette-Guerin (BCG) is the only FDA approved first line therapy for patients with nonmuscle invasive bladder cancer. The purpose of this study is to better understand the role of innate immune pathways involved in BCG immunotherapy against murine bladder tumor. We first characterized the immunological profile induced by the MB49 mouse urothelial carcinoma cell line. MB49 cells were not able to activate an inflammatory response (TNF-α, IL-6, CXCL-10 or IFN-β) after the stimulus with different agonists or BCG infection, unlike macrophages. Although MB49 cells are not able to induce an efficient immune response, BCG treatment could activate other cells in the tumor microenvironment (TME). We evaluated BCG intratumoral treatment in animals deficient for different innate immune molecules (STING^−/−^, cGAS^−/−^, TLR2^−/−^, TLR3^−/−^, TLR4^−/−^, TLR7^−/−^, TLR9^−/−^, TLR3/7/9^−/−^, MyD88^−/−^, IL-1R^−/−^, Caspase1/11^−/−^, Gasdermin-D^−/−^ and IFNAR^−/−^) using the MB49 subcutaneous mouse model. Only MyD88^−/−^ partially responded to BCG treatment compared to wild type (WT) mice, suggesting a role played by this adaptor molecule. Additionally, BCG intratumoral treatment regulates cellular infiltrate in TME with an increase of inflammatory macrophages, neutrophils and CD8+ T lymphocytes, suggesting an immune response activation that favors tumor remission in WT mice but not in MyD88^−/−^. The experiments using MB49 cells infected with BCG and co-cultured with macrophages also demonstrated that MyD88 is essential for an efficient immune response. Our data suggests that BCG immunotherapy depends partially on the MyD88-related innate immune pathway.

## Introduction

Advances in immunotherapy are extremely promising with new possibilities for anti-tumor therapy comprising vaccines, antibodies, checkpoint inhibitors, CAR-T cells and even viruses or bacteria to activate the immune response^1^. Although most of these are very recent technologies, this concept has been applied for more than 40 years in bladder cancer using the BCG (Bacillus of Calmette-Guérin) vaccine as an efficient immunotherapy. Currently, the use of intravesical BCG immunotherapy to reduce the risk of recurrence and progression^2^ in patients with non-muscle invasive bladder cancer after transurethral resection (TURBT) is recommended by the most important international associations^3,4^. Despite the efficiency of BCG immunotherapy, some patients have side effects, relapses and even resistance to BCG and about 30% of patients do not respond to treatment^5^. Although BCG has been applied for long time in the treatment of bladder cancer there is still many points to address to better understand its mechanisms of action.

The result of immunotherapy depends not only on the response from the immune cells, but also the intrinsic response from the tumor cells. Bladder cancer cells and also benign urothelial cells play an initial role in the recognition and processing of BCG, for later activation of the immune response and subsequent regulation of bladder microenvironment resulting in cytotoxicity against cancer cells^6,7^. TLRs (Toll-like receptors) act as an important interface between innate and adaptive immunity. The mycobacteria lipoproteins are recognized as PAMP (pathogen-associated molecular pattern) by TLR2 and TLR9 recognizes bacterial DNA^8–10^. TLR4 signaling does not seem to be essential in the initial control of BCG infection, but it is required to activate a robust Th1 response^9–12^. Therefore, TLRs play an important role in the response to BCG immunotherapy and many TLR agonists have been considered as candidates for bladder cancer therapy^5,13–15^.

cGAS-STING pathway is another important mechanism of the innate immunity for cytosolic DNA recognition, promoting the activation of TBK1 and phosphorylation of IRF-3, a transcription factor that induces the expression of IFN-β^16^. *Mycobacterium tuberculosis* and *M. bovis* (BCG) induce the production of IFN-β depending on the detection of c-di-AMP (cyclic-di-adenosine monophosphate) and activation of the STING pathway, which leads to the increase of autophagy in macrophages and bacterial control in mice^17^. STING is involved in the antitumor immune response playing an essential role in the recognition of cancer cells and in the activation of type I IFN-dependent cytotoxic T cell response^18,19^. The therapeutic intratumoral administration of cGAMP (cyclic dinucleotide GMP-AMP) or CDNs (cyclic di-nucleotides) suppresses tumor growth, presumably through the direct activation of STING in the tumor microenvironment (TME), leading to the activation of phagocytic cells-dependent cytotoxic T lymphocytes response^20–22^. STING also plays an important role in the antitumor effects associated with chemotherapeutic agents^23^ and is essential for the immunotherapeutic response of radiation-induced T cells^24,25^. However, the role of the cGAS-STING pathway and IFN-β in BCG immunotherapy have been poorly investigated.

Tumor immunotherapy depends on the cell profile present in TME, which can promote a predominantly pro- or anti-inflammatory response. This initial innate immune response in the TME is mainly induced by dendritic cells and macrophages (tumor-associated macrophages—TAM), which may present a polarization type M1 (pro-inflammatory, anti-tumor) or M2 (anti-inflammatory, pro-tumor), essential for the recruitment of lymphocytes to the tumor infiltrate (tumor-infiltrating lymphocytes—TIL) and the activation of an efficient adaptive immune response^26,27^. M2 macrophages-profile predominance in bladder tumors infiltrate is related to a worse prognosis and BCG immunotherapy failure^28–30^. Signaling via type I IFN in tumors is crucial for the recruitment of lymphocytes in the TME and several studies seek to activate this route to potentiate the anti-tumor response^20,31,32^.

The understanding of the possible mechanisms involved in BCG immunotherapy can guide the selection of specific therapies for each patient and bring new alternatives to replace or associate with existing therapies. Thus, this study aimed to investigate the involvement of the cGAS-STING pathway, different TLRs (TLR2, TLR3, TLR4, TLR7 and TLR9) and MyD88 adaptor protein in the immunotherapy with BCG to treat MB49 syngeneic mouse bladder cancer. Even though MB49 tumor cells did not efficiently activate an immune response, BCG presence in bladder cancer could induce the appropriate response from other immune cells present in the TME. We evaluate BCG intratumoral treatment in a subcutaneous MB49 tumor model using different knockout (KO) mice (STING^−/−^, cGAS^−/−^, TLR2^−/−^, TLR3^−/−^, TLR4^−/−^, TLR7^−/−^, TLR9^−/−^, TLR3/7/9^−/−^, MyD88^−/−^, IL-1R^−/−^, Caspase1/11^−/−^, Gasdermin-D^−/−^ and IFNAR^−/−^). MyD88 was the only molecule relevant for tumor regression after BCG treatment in vivo and also involved in activation of macrophages in co-culture with infected MB49. Additionally, this study show the importance of MyD88 regulating cellular infiltrate and inflammatory profile in TME during BCG treatment. In summary, our report suggests the involvement of MyD88 and not a single TLR tested here in BCG immunotherapy against bladder cancer.

## Results

### BCG treatment reduces subcutaneous bladder tumor

**Figure 1:**
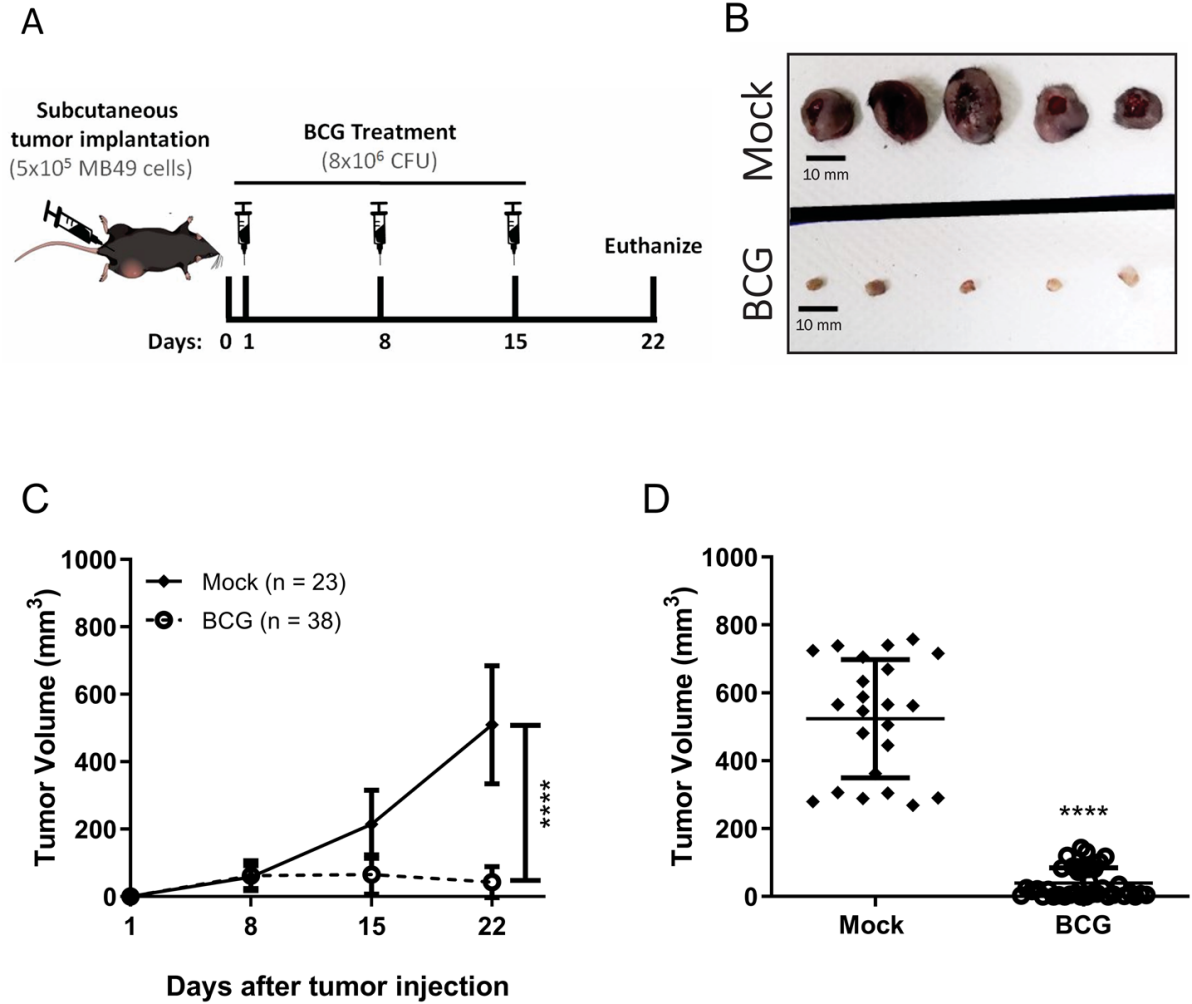
BCG treatment in subcutaneous bladder tumor. (**A**) Scheme of the BCG treatment in a heterotopic (subcutaneous) syngeneic tumor model with MB49 bladder tumor cells in female C57BL/6 mice. MB49 cells (5 × 10^5^ cells) were subcutaneously injected on the right flank. After 24 h of tumor injection, mice received the first intratumoral treatment with BCG (8 × 10^6^ CFU) or PBS (Mock). The treatments were performed every 7 days (total three doses) and the tumor volume was evaluated once a week using a digital caliper. Mice were euthanized and the tumors removed after 22 days post-tumor injection. (**B**) Representative image of the BCG or Mock treatment effect in subcutaneous bladder tumor (Scale bar, 10 mm). Tumor growth curves (**C**) and the final volumes after 22 days (**D**) are shown. Figures (**C**) and (**D**) represents the tumor volumes mean and standard deviation summary of C57BL/6 WT control animals from the following in vivo experiments comparing WT and KO mice. ****Statistically significant compared to mock, *P* ≤ 0.0001.

The effect of BCG treatment on mice subcutaneous administered with MB49 bladder tumors has already been demonstrated in several studies. The different BCG strains are characterized by phenotypic and immunogenic differences with a variable of virulence levels, leading to different anti-tumor response^33^. We compared BCG Moreau and BCG Pasteur and both strains show very similar effects in tumor volume reduction (Supplementary Fig. S1). So, we decided to use BCG Moreau because the strain is the most commonly used in Brazil and the virulence is considered intermediary^34^. Tumor regression effect is more evident when the treatment starts right after the tumor injection than after the tumor grow^35^. In addition, bladder tumors development depends on the hormone testosterone, which may explain why bladder cancer is more prevalent in men than women and MB49 tumor grows rapidly in male than in female mice^36^. After some trials, we established the heterotopic syngeneic model by the subcutaneous injection of MB49 bladder cancer cells (5 × 10^5^ cells) in female wild type C57BL/6 (WT) and different knockout mice (KO). After 24 h post-tumor injection, animals were treated intratumorally with BCG (8 × 10^6^ CFU) or PBS (mock) every seven days (total three doses) (Fig. 1A). After 22 days post-tumor injection we euthanized the animals, dissected the tumors and observed that BCG treatment robustly reduced tumor size compared to mock (Fig. 1B). BCG treatment shows partial or total reduction in tumor volume reinforcing the efficacy of the immunotherapy (Fig. 1C,D).

### Innate immune signaling pathways are impaired in MB49 cells

**Figure 2:**
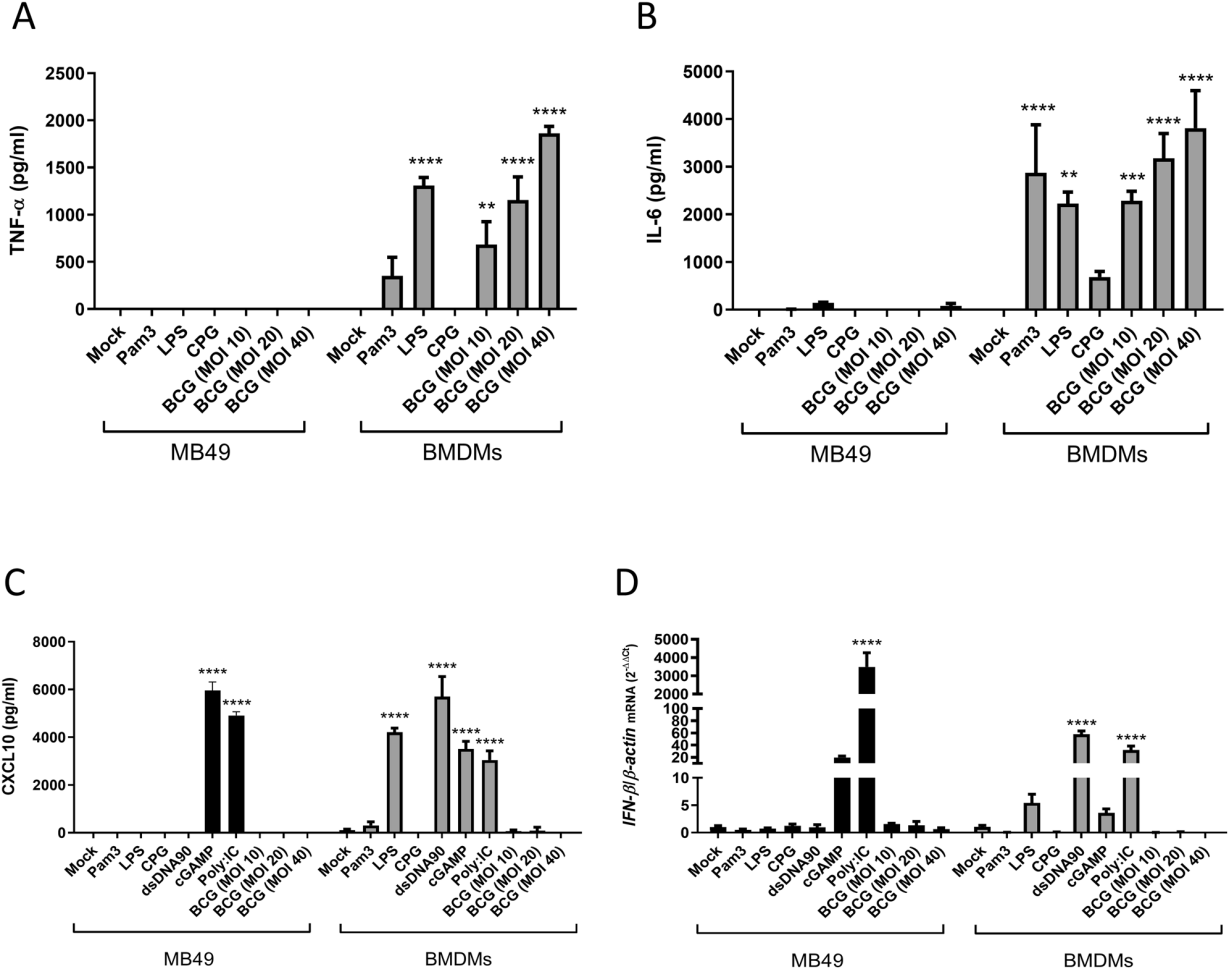
Activation of several innate immune pathways is impaired in MB49 cells. MB49 tumor cells and bone marrow-derived macrophages (BMDMs) were stimulated with Pam3CSK4 (1 µg/ml), ultrapure LPS (100 ng/ml), CpG-ODN (1 µg/ml) or BCG (MOI 10, 20 or 40). MB49 and BMDM cells were transfected with PolyI:C (3 µg/ml), dsDNA90 (3 µg/ml) or cGAMP (6 µg/ml) in addition to Lipofectamine 2000 (3ug/ml). The production of TNF-α (**A**), IL-6 (**B**) and CXCL10 (**C**) in cell supernatants was analyzed after 24 h of stimulus by ELISA. *IFN-β* expression after 1 h was determined by qPCR (**D**). All qPCR results were relative to *β-actin* mRNA as a normalizer. MB49 unstimulated sample (Mock) or BMDM (Mock) were used as control. The values are representative of at least two independent experiments. *Statistically significant compared to mock from the same cell line, **P ≤ 0.01, ***P ≤ 0.001, ****P ≤ 0.0001.

The innate immune pathways are essential for the activation of adaptive immunity and to provide an efficient immune response against the tumor. However, we know that many tumor cell lines are deficient for some of these pathways. For example, it has already been demonstrated that cGAS-STING pathway is deficient in different tumor cell lines due to the inhibition of cGAS and/or STING expression by a process of DNA hypermethylation^37–40^. In order to evaluate the activation of some different innate immune signaling pathways in MB49 cells, we compared MB49 to C57BL/6 bone marrow-derived macrophages (BMDMs) that are well known for its capacity to strongly respond after stimulation with different agonists and BCG infection. Although we verified the production of inflammatory cytokines such as TNF-α (Fig. 2A) and IL-6 (Fig. 2B) in BMDMs activated with Pam3, LPS, CpG or BCG, the same was not detected in MB49 cells which did not respond to any of these agonists. This result suggests that TLR2, TLR4 and TLR9 receptor innate immune pathways are impaired in MB49 cells. In addition, we also evaluate the activation of pathways that recognize nucleic acids, such as cGAS-STING, TLR3 and RIG-I. Surprisingly, when MB49 was activated with PolyI:C via TLR3 and/or RIG-I these cells were capable to produce large amounts of CXCL-10 and to express high levels of *IFN-β* (Fig. 2C,D). The cGAS-STING pathway was not activated in MB49 in the presence of dsDNA, but responded to direct stimulus of cGAMP producing CXCL10 and expressing *IFN-β*, which suggests that cGAS is not active in this tumor cell line (Fig. 2C,D). BCG infection (MOI 10, 20 and 40) was unable to directly activate MB49 to produces any of the cytokines measured (Fig. 2A–D), demonstrating the ineffectiveness of MB49 cells to activate some of the innate immune pathways.

### Spleen cells from animals treated with BCG are strongly activated in co-culture with infected MB49 cells

**Figure 3:**
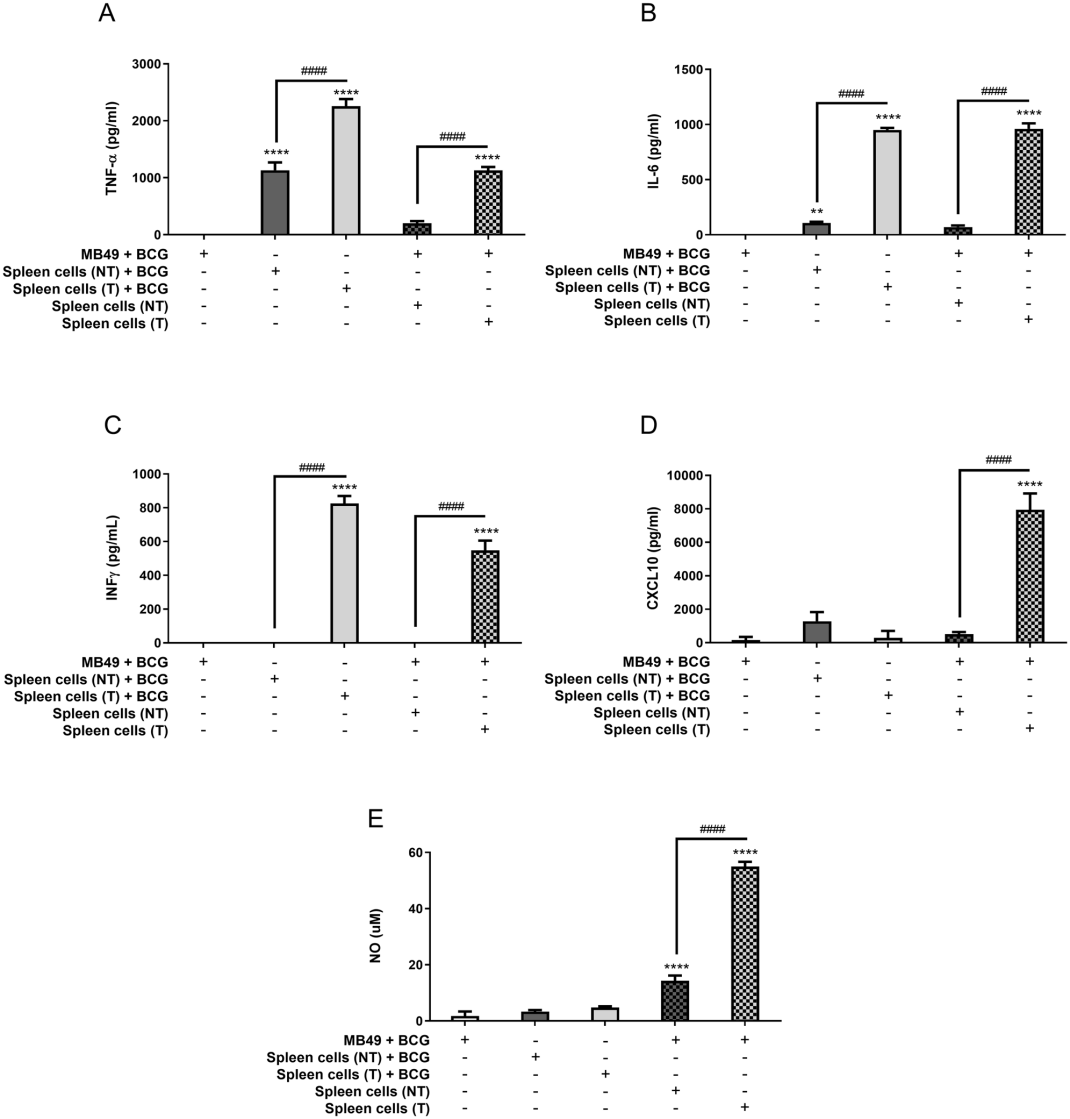
Spleen cells from animals treated with BCG are strongly activated in co-culture with infected MB49 cells. Spleen cells were obtained from mice submitted to the subcutaneous tumor model and treated with BCG (T) or control PBS (not treated—NT) (as shown in Fig. 1A) and euthanized at day 22 post-tumor injection. Splenocytes suspension was used for co-culture in vitro with infected MB49 cells (1 MB49 cell: 2 spleen cells). MB49 cells used for co-culture were previously infected with BCG (MB49 + BCG) for 24 h and washed to remove free BCG in the supernatant. Co-cultured cells were maintained together for 24 h (**A**–**D**) or 48 h (**E**), when the samples were collected for analysis. MB49 or splenocytes were also infected separately (not in co-culture) and used as controls. Measurement of TNF-α (**A**), IL-6 (**B**), IFN-γ (**C**) and CXCL10 (**D**) production in the supernatants after 24 h was performed by ELISA. (**E**) NO production was evaluated by the Griess method in supernatants after 48 h of stimulation. The values are representative of at least three independent experiments. *Statistically significant compared to MB49 + BCG, **P ≤ 0.01, ****P ≤ 0.0001. ^#^Statistically significant data comparing treated (T) and not treated (NT), ^####^*P* < 0.0001.

Since MB49 cells demonstrated an inability to be activated in response to different stimulus, we addressed whether BCG was able to infect these cells. Then, we infected MB49 cells with BCG (MOI 5, 10, 20 e 40) for 6, 24 and 48 h and evaluated intracellular bacteria CFU counts. The numbers of intracellular bacteria were similar in all time points tested confirming that BCG was able to infect MB49 cells (Supplementary Fig. S2A). Additionally, MB49 infected cells (BCG MOI 10, 20 and 40) release similar amounts of lactate dehydrogenase (LDH) in the supernatant, suggesting that BCG infection was not causing cell death (Supplementary Fig. S2B). Since MB49 cells were infected but not directly activating the innate immune response, we hypothesized that the tumor cells could be activating other immune cells present in TME and generating a systemic response. To evaluate this hypothesis, we proceeded with some experiments co-culturing previously infected MB49 cells with spleen cells from mice that received subcutaneous implantation of MB49 tumors and treated with BCG (treated—T) or control PBS (not treated—NT). These animals were euthanized 21 days after tumor injection to remove the spleens and prepare the total splenocytes. MB49 cells used for co-culture were previously infected (BCG MOI 40) for 24 h and washed to remove free BCG in the supernatant, followed by the addition of the splenocytes and maintained in co-culture for 24 or 48 h. MB49 cells and splenocytes separated (not in co-culture) were infected at the same time as the co-culture and maintained as controls. The amount of intracellular BCG in MB49, spleen cells or co-cultured cells were very similar even in the spleen cells from BCG treated mice (Supplementary Fig. S2C). Regarding cytokine production, our data reinforces that MB49 does not respond to BCG stimulation (Fig. 3A–C). Instead, we observed the production of TNF-α, IL-6 and IFN-γ in response to BCG infection both in the splenocytes only and in the co-cultured MB49 with splenocytes. The cytokine response detected was stronger in spleen cells from mice that were intratumorally treated with BCG. In contrast, we detected higher levels of CXCL10 and NO only in co-culture of infected MB49 and splenocytes from BGC treated mice (Fig. 3D,E). Cells in co-culture without BCG infection were not activated (data not shown). The co-culture data indicates that tumor treatment with BCG leads the spleen cells from these mice to a more responsive state. The immune response activated from BCG in the spleen cells could favor a systemic antitumor response in mice.

### Successful BCG treatment against subcutaneous bladder tumor is independent of cGAS-STING pathway and TLR3 receptor

**Figure 4:**
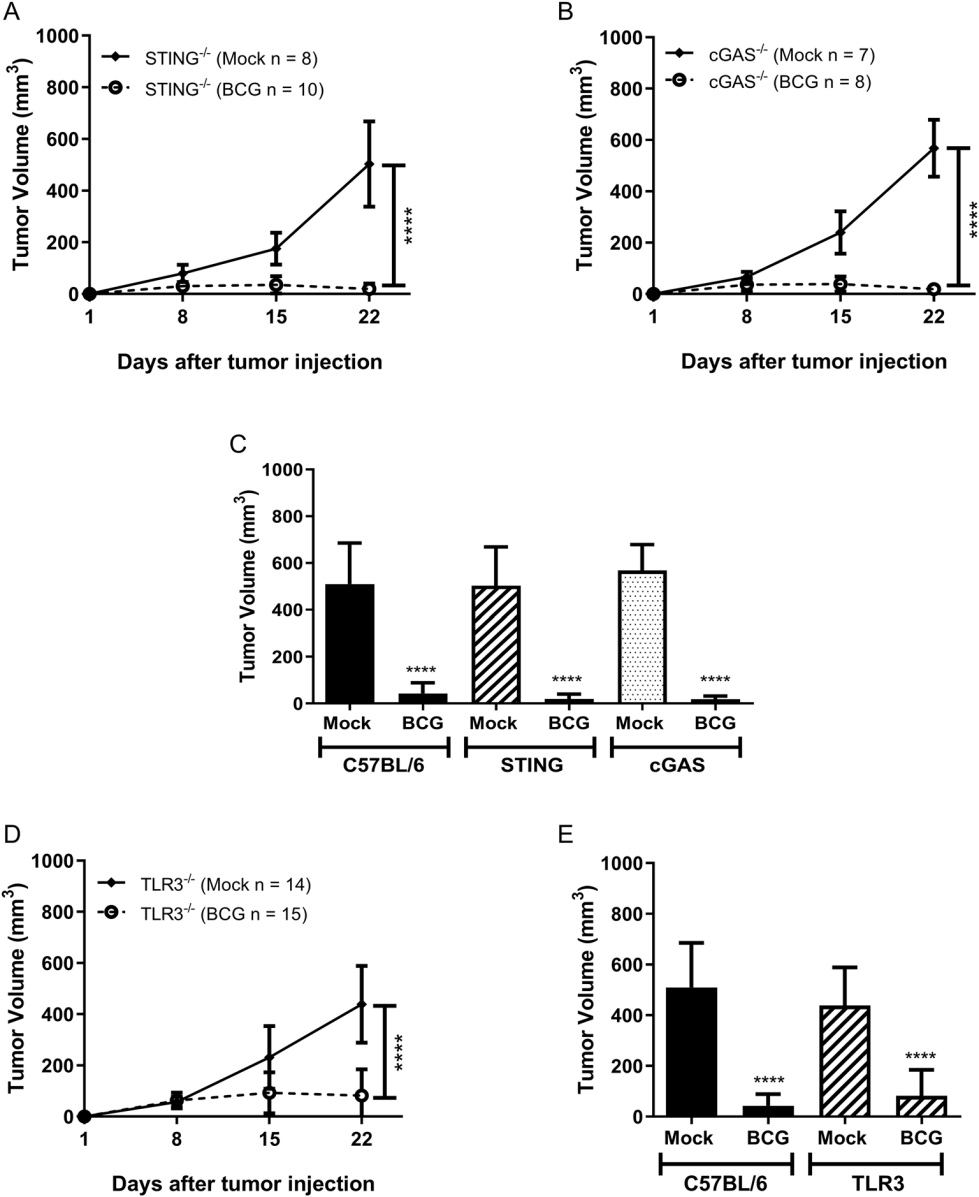
The effect of BCG treatment on subcutaneous tumor is independent of cGAS-STING and TLR3 signaling pathways. C57BL/6 WT, STING^−/−^, cGAS^−/−^ and TLR3^−/−^ mice were submitted to the subcutaneous tumor model injection and BCG treatment. Tumor growth curves are shown for STING^−/−^ (**A**), cGAS^−/−^ (**B**) and TLR3^−/−^ (**D**). The final volumes (day 22) comparing C57BL/6 WT with STING^−/−^ and cGAS^−/−^ animals are shown in Figure (**C**) and C57BL/6 WT compared to TLR3^−/−^ in Figure (**E**). Data represents mean and standard deviation from the results of at least two independent experiments. C57BL/6 WT data represents the summary of control animals from all in vivo experiments comparing WT and KO mice. *Statistically significant compared to the respective untreated control, ****P ≤ 0.0001.

In order to evaluate the innate immune pathways related to BCG tumor treatment, we tested the MB49 syngeneic tumor model in different KO mice. The cGAS-STING pathway is activated by double-stranded DNA in the cytoplasm and is well known for its importance in antiviral response, inflammation and cancer. cGAS works in concert with STING adaptor molecule to trigger an innate immune response^41^. BCG intratumoral treatment in cGAS^−/−^ and STING^−/−^ mice presents similar results as C57BL/6 WT animals, discarding the importance of this pathway in BCG tumor treatment (Fig. 4A–C). TRIF molecule is essential for TLR3 signaling pathway, a sensor for double-stranded RNA, important to mediate regulation in the pathogenesis of mycobacterial infection^42^. TLR3^−/−^ mice did not show difference in BCG tumor treatment compared to WT (Fig. 4D,E). Therefore, the effect of BCG treatment on bladder tumors is independent of TLR3 and the cGAS-STING pathway.

### The MyD88 adaptor molecule is essential for BCG-dependent tumor regression

**Figure 5:**
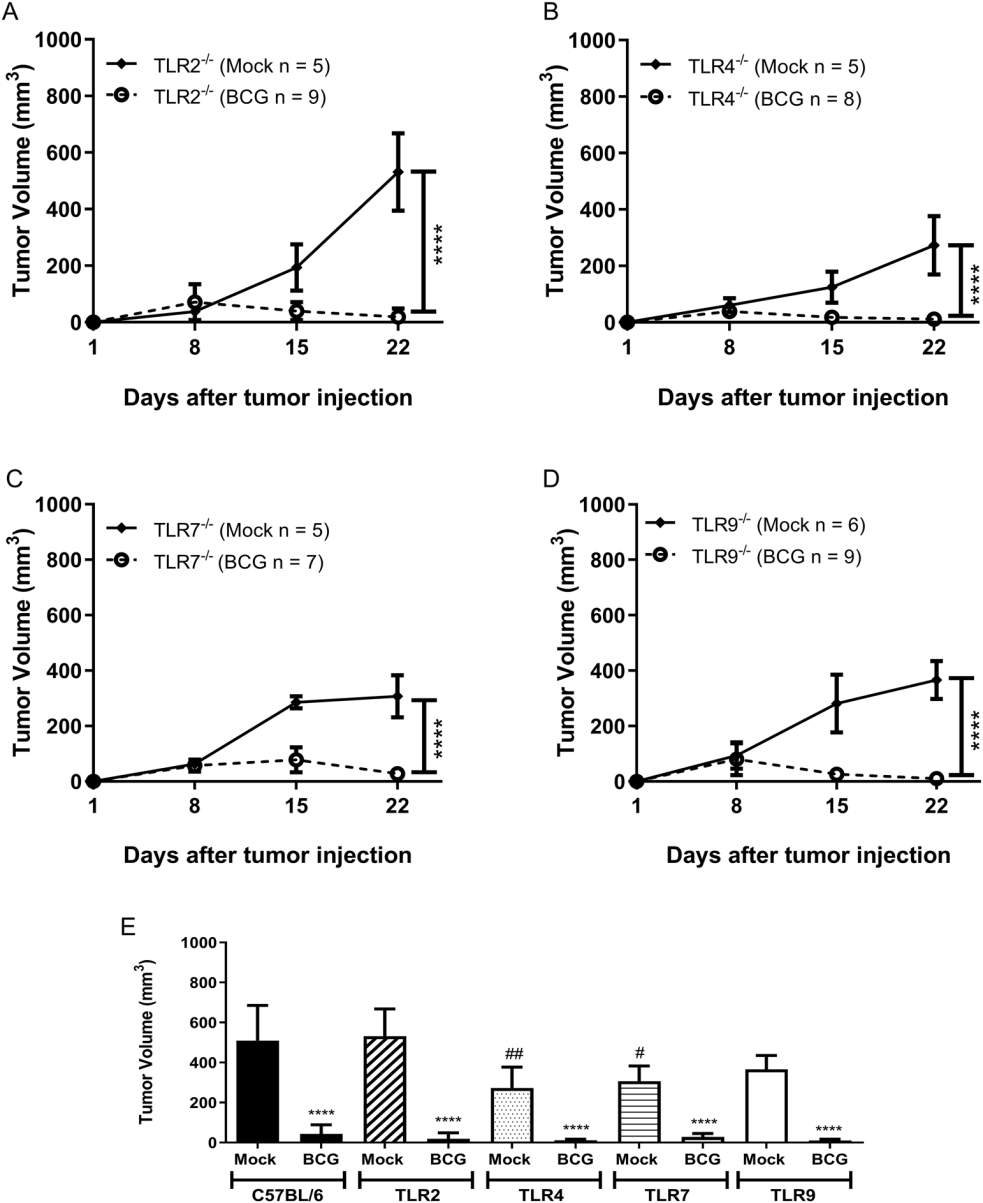
TLR2, TLR4, TLR7, and TLR9 receptors do not interfere in tumor regression in response to BCG treatment. C57BL/6 WT, TLR2^−/−^, TLR4^−/−^, TLR7^−/−^ and TLR9^−/−^ mice were submitted to the subcutaneous tumor model and BCG treatment. Tumor growth curves are shown for TLR2^−/−^ (**A**), TLR4^−/−^ (**B**), TLR7^−/−^ (**C**) and TLR9^−/−^ (**D**). The final volumes (day 22) are shown in Figure (**E**). Data represents mean and standard deviation from the results of at least two independent experiments. C57BL/6 WT data represents the summary of control animals from all in vivo experiments comparing WT and KO mice. *Statistically significant compared to the respective untreated control, ****P ≤ 0.0001. ^#^Statistically significant compared to C57BL/6 WT mock not treated, ^#^*P* ≤ 0.05, ^##^*P* ≤ 0.01, ^####^*P* ≤ 0.0001.

**Figure 6:**
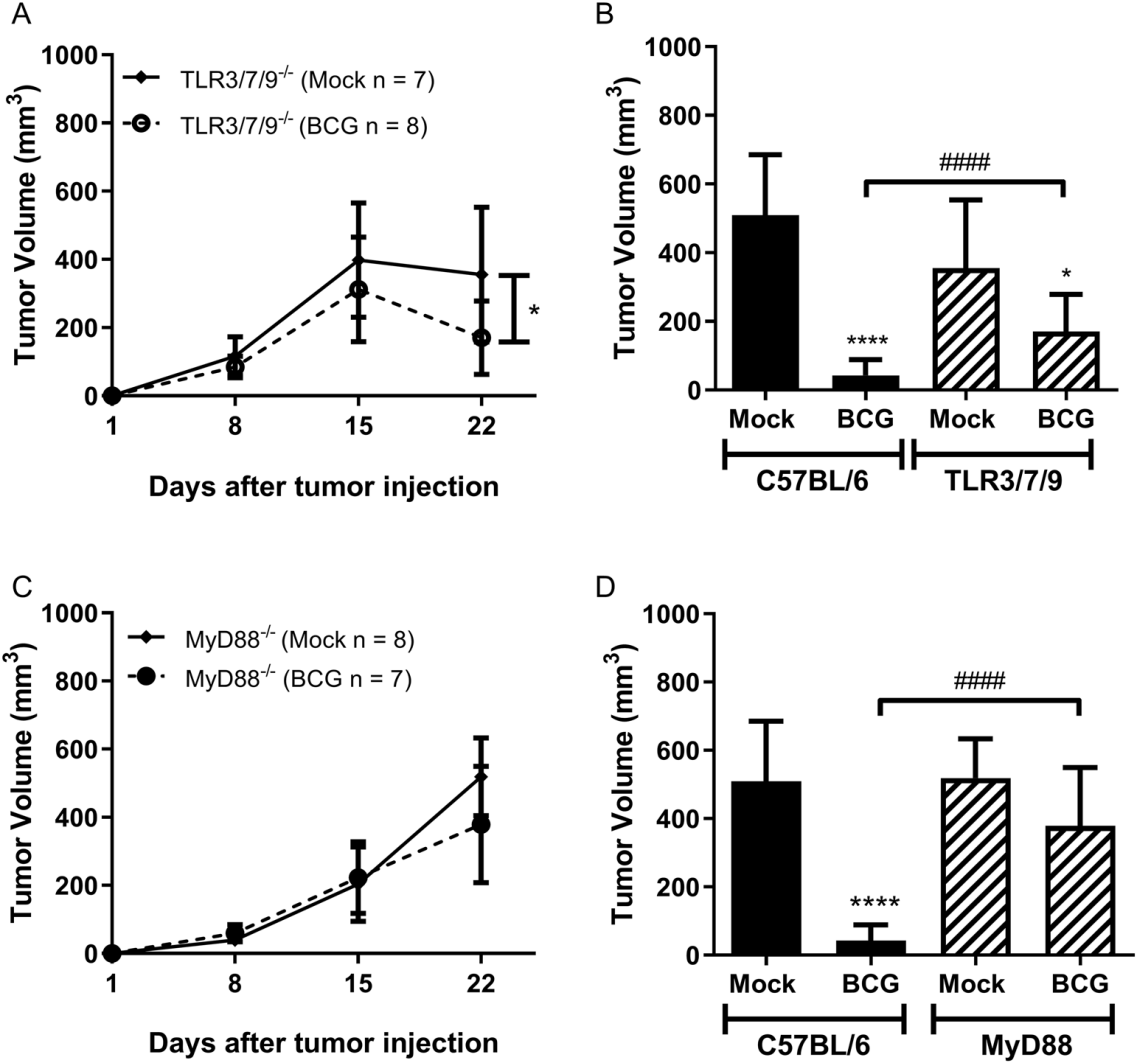
Tumor treatment with BCG is partially dependent on a synergistic effect of endosomal TLRs and totally dependent on MyD88 signaling. Triple deficient animals TLR3/7/9^−/−^ were used to evaluate the synergistic effect of endosomal TLRs and MyD88^−/−^ deficient mice used to define the role of this adaptor molecule in tumor regression. Mice were submitted to the subcutaneous tumor model and BCG treatment. Tumor growth curves and the final volumes (day 22) are shown for TLR3/7/9^−/−^ (**A**, **B**) and MyD88^−/−^ (**C**, **D**). Data represents mean and standard deviation from the results of at least two independent experiments. C57BL/6 WT data represents the summary of control animals from all in vivo experiments comparing WT and KO mice. *Statistically significant compared to the respective untreated control, *P < 0.05, ****P ≤ 0.0001. ^#^Statistically significant compared to BCG treated C57BL/6 WT, ^####^*P* ≤ 0.0001.

MyD88 adaptor is critical for the majority of the TLRs signaling. Specially TLR2, TLR4 and TLR9 are strongly related to the immune response against BCG. Herein, we investigate the effect of BCG tumor treatment in TLR2^−/−^ (Fig. 5A), TLR4^−/−^ (Fig. 5B), TLR7^−/−^ (Fig. 5C) and TLR9^−/−^ mice (Fig. 5D). All four TLRs KO mice responded similarly to WT regarding BCG tumor treatment (Fig. 5E). In this study, TRIF- or MyD88-dependent single TLRs did not show any relevance for BCG tumor treatment. Therefore, our strategy was to investigate the synergistic effect of a triple deficient TLR3/7/9^−/−^ animals eliminating the endosomal TLRs signaling or MyD88^−/−^ deficient mice used to abrogate completely all the receptors pathways related to MyD88. TLR3/7/9^−/−^ mice showed a delay in tumor regression and only a partial tumor volume reduction compared to WT mice (Fig. 6A,B). The removal of the adaptor molecule MyD88 completely eliminated the effect of BCG in tumor volume reduction (Fig. 6C,D). Complementary studies with IL-1R^−/−^, caspase1/11^−/−^ and Gasdermin-D^−/−^ demonstrated that MyD88 role in BCG treatment does not involve specifically the activation of the inflammasome pathway (Supplementary Fig. S3A–D). Tumor growth experiments using interferon receptor knockout mice (IFNAR^−/−^) were performed and we observe that BCG tumor treatment is independent on type I IFN signaling (Supplementary Fig. S3E,F). The results suggest that there is a synergistic effect of several receptors that signal via the MyD88-dependent pathway favoring the action of BCG in tumor treatment.

### BCG treatment alters the immune cells infiltrate profile in TME

**Figure 7:**
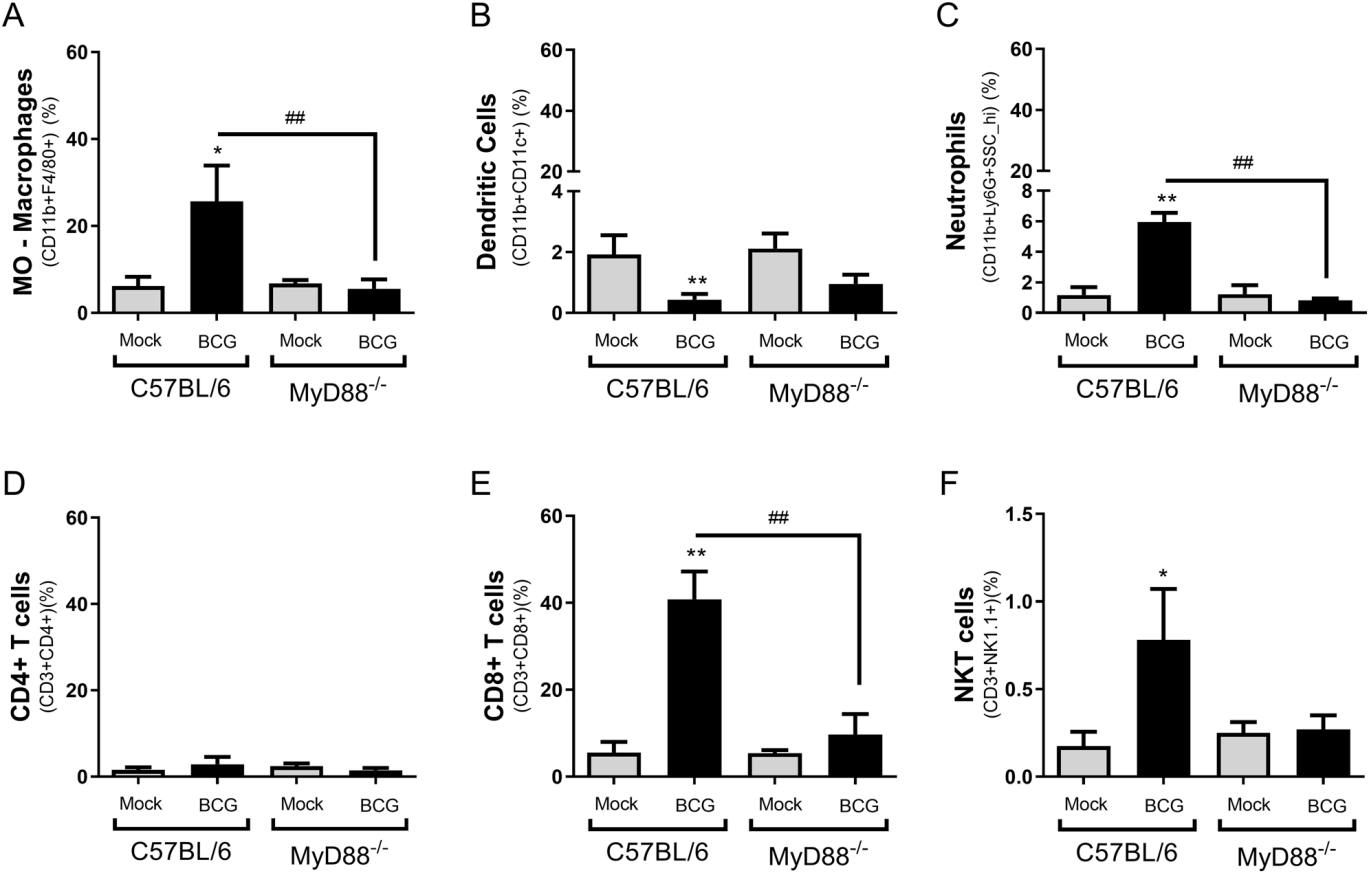
BCG treatment alters the immune cells infiltrate profile in TME and induces MyD88-dependent inflammatory activation. MB49 tumor cells (5 × 10^5^ cells) were implanted subcutaneously in C57BL/6 WT or MyD88^−/−^ mice. These animals were intratumorally treated with two doses of BCG (8 × 10^6^ CFU) or PBS (mock) and they were euthanized 15 days after the tumor injection. Tumors were dissected, micend and the cells were dissociated with collagenase IV (200 U/ml). Cell infiltrate was analyzed by flow cytometry using specific markers to: macrophages (CD11b + F4/80 +) (**A**); dendritic cells (CD11b + CD11c +) (**B**); neutrophils (CD11b + Ly6G + SSC hi) (**C**); CD4 T lymphocytes (CD3+ CD4+) (**D**); CD8 T lymphocytes (CD3+ CD8+) (**E**); NKT cells (CD3 + NK1.1 +). The graphs represent the cell percentage relative to total cell population contained in TME. The results are representative of at least two independent experiments. *Statistically significant compared to the respective untreated control, **P ≤ 0.01, ***P ≤ 0.001, ****P ≤ 0.0001. ^#^Statistically significant comparing BCG treated C57BL/6 WT and MyD88^−/−^, ^#^*P* ≤ 0.05, ^###^*P* ≤ 0.001, ^####^*P* ≤ 0.0001.

**Figure 8:**
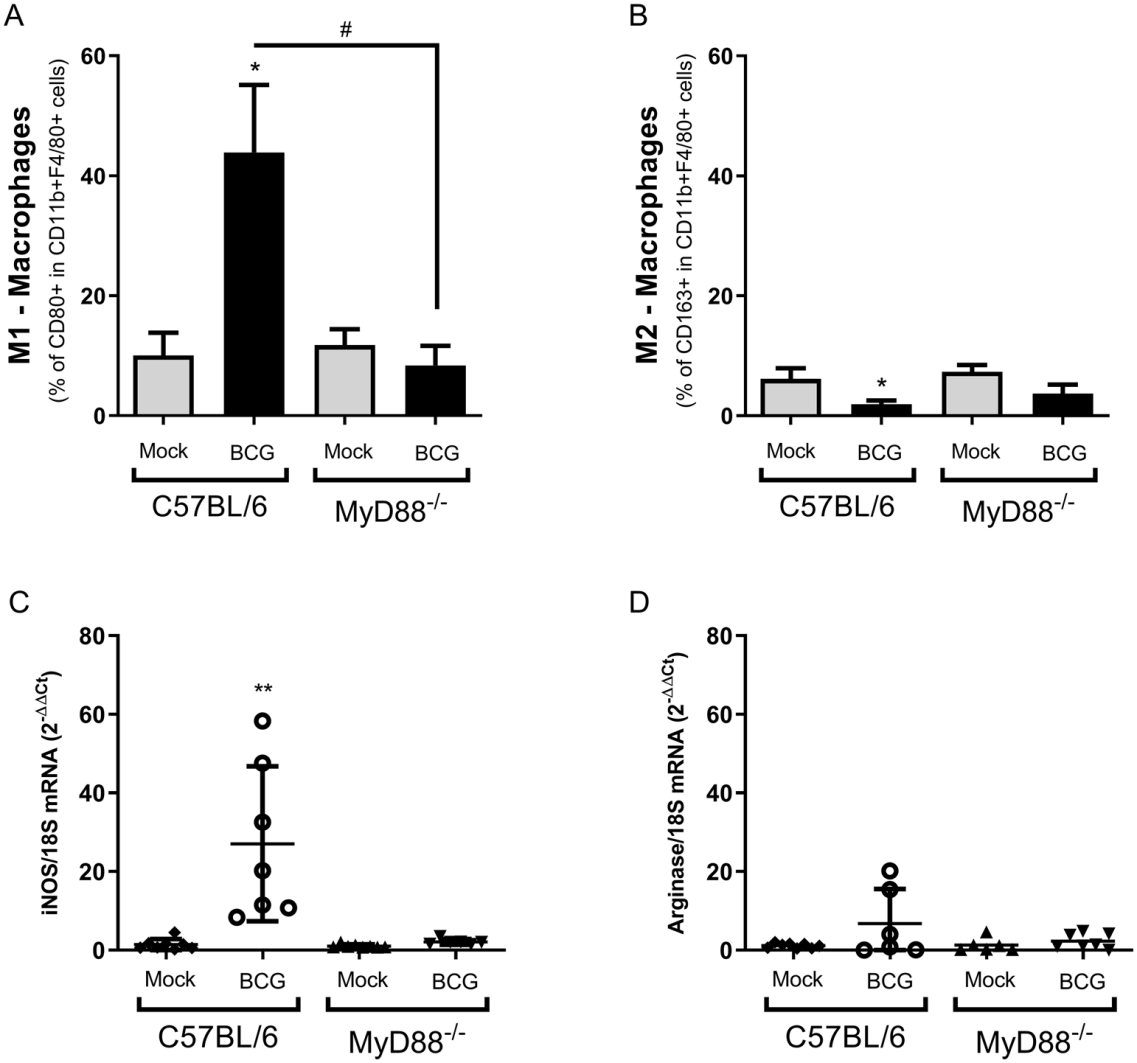
The inflammatory macrophages profile present in TME from tumors treated with BCG. Fifteen days syngeneic tumors from C57BL/6 WT or MyD88^−/−^ mice (same as used in Fig. 7) were evaluated concerning activation status of macrophages. Cell infiltrate was analyzed by flow cytometry using specific markers to address M1 (CD11b + F4/80 + CD80 +) (**A**) and M2 (CD11b + F4/80 + CD163 +) (**B**) macrophages profiles. The graphs percentages are relative to CD11b + F4/80+ (Macrophages) entire population. The mRNA expression in the same group of tumors was also analyzed by qPCR for the following targets: iNOS (**C**) and Arginase (**D**). All qPCR results were relative to 18S mRNA as normalizer. Untreated (mock) tumors from each group were used as control. Data represents ΔΔCT mean and standard deviation from tumors in two independent experiments. *Statistically significant compared to the respective untreated control (mock), *P < 0.05, ***P ≤ 0.001. ^#^Statistically significant comparing BCG treated C57BL/6 WT and MyD88^−/−^, ^#*##*^*P* ≤ 0.001.

In order to evaluate the importance of MyD88 to influence TME, we compared tumors from C57BL/6 WT and MyD88^−/−^ mice after two BCG intratumoral treatments (15 days). Tumors from euthanized mice were dissected, dissociated using collagenase IV and the cell infiltrate was analyzed by flow cytometry using specific markers to different immune cells. Intratumoral BCG treatment in WT mice regulates the cellular infiltrate in TME with a significant increase in the percentage of macrophages (Fig. 7A), neutrophils (Fig. 7C), CD8+ T lymphocytes (Fig. 7E) and NKT cells (Fig. 7F) population compared to MyD88^−/−^ animals. No relevant difference in dendritic cells (Fig. 7B) and CD4+ T lymphocytes (Fig. 7D) population was observed in MyD88^−/−^ and WT mice treated with BCG. The TME was also evaluated concerning the activation status of macrophages. A significant increment in the population of inflammatory macrophages (M1) was observed in tumors treated with BCG only from WT mice but not MyD88^−/−^, a change not observed for type 2 macrophages (M2), which presented a very low percentage of CD163^+^ cells in macrophages from all mouse groups tested (Fig. 8A,B). We also detected an upregulation of *iNOS* mRNA expression unlike arginase (Fig. 8C,D) after two BCG treatments in tumors from WT mice. All the analysis from TME indicates an increase in cell infiltrate and cell activation in WT mice, but not in MyD88^−/−^, favorable to tumor remission.

### Macrophages are essential for the immune response during BCG tumor treatment

**Figure 9:**
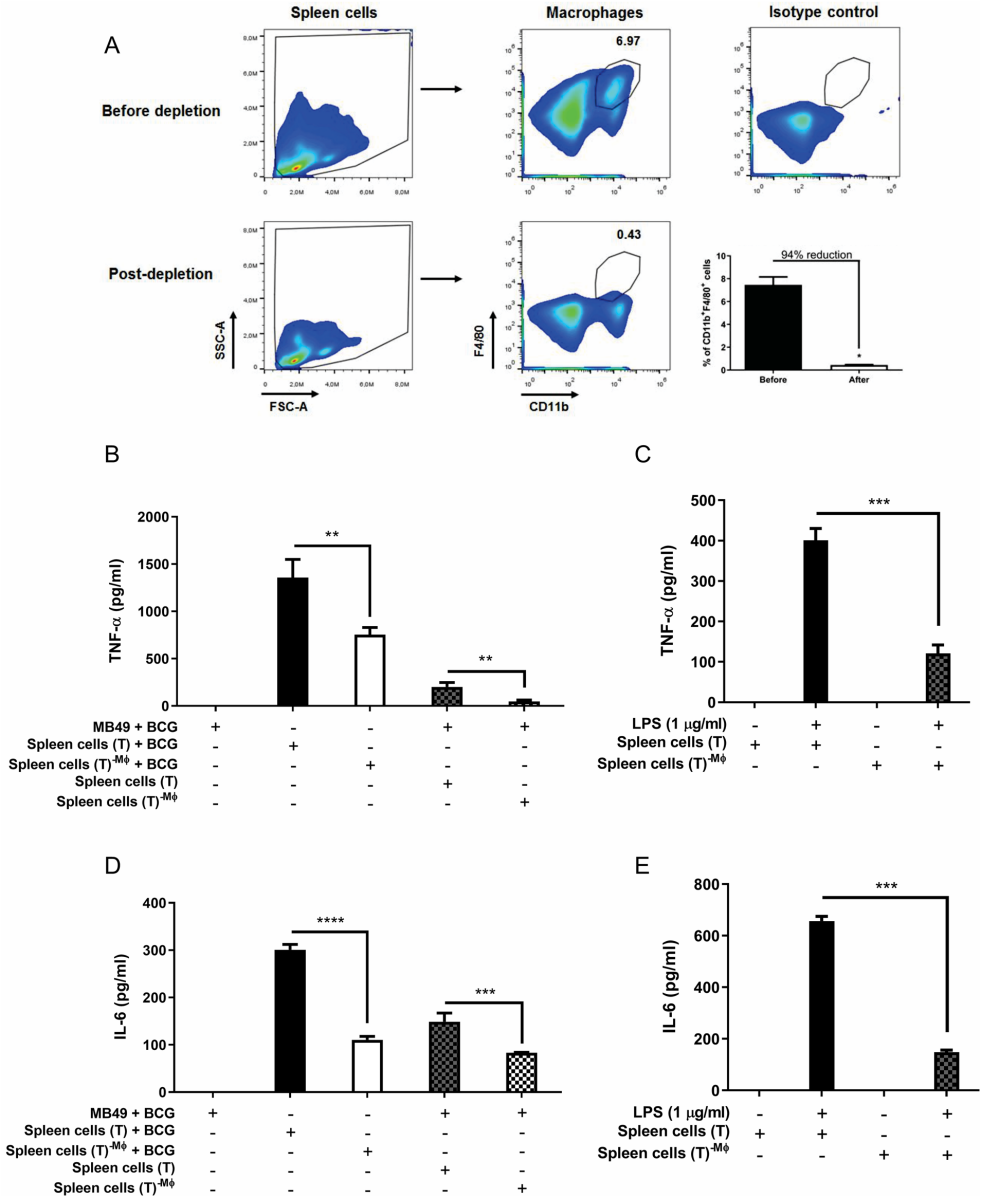
Co-culture performed with macrophages-depleted spleen cells reduces proinflammatory cytokines production. Spleen cells were obtained from mice submitted to the subcutaneous tumor model and treated with BCG (T) after 22 days post-tumor injection. CD11b^+^F4/80^+^ cells (Macrophages—MØ) were depleted from the spleen cells (T) using anti-PE MicroBeads kit described in the methodology. The percentage of depletion (94%) was addressed by flow cytometry. (**A**) A representative plot and frequency are shown. Total spleen cells (T) or macrophages-depleted spleen cells (T)^−MØ^ suspension were used for co-culture in vitro with infected MB49 cells (1 MB49 cell: 2 spleen cells). MB49 cells used for co-culture were previously infected with BCG (MB49 + BCG) for 24 h and washed to remove free BCG in the supernatant. (**B**, **D**) Co-cultured cells were maintained together for 24 h. MB49 or spleen cells were also infected separately (not in co-culture) and used as controls. (**C**, **E**) Spleen cells were also stimulated with LPS (1 µg/ml) for 24 h as controls. TNF-α (**B**, **C**) and IL-6 (**D**, **E**) cytokines production were measured in the supernatants after 24 h by ELISA. The values are representative of two independent experiments. *Statistically significant compared to spleen cells (T) before macrophage depletion. **P ≤ 0.01, ***P ≤ 0.001, ****P ≤ 0.0001.

In order to confirm the importance of macrophages we performed co-culture experiments with macrophages-depleted spleen cells from mice previously treated intratumorally with BCG. After standard intratumoral treatment with BCG for 3 weeks, C57BL/6 WT mice were euthanized for spleen cells suspension preparation as we explained previously. Cells were submitted to a depletion protocol using magnetic beads to negatively select macrophages previously labeled with anti-PE antibody. Cells remaining after depletion were quantified by flow cytometry and we determined a reduction of 94% in macrophages population among the spleen cells. We compared the co-culture of MB49 with spleen cells before and after macrophages depletion. The results show a reduction on TNF-α and IL-6 cytokines after depletion and reinforce that macrophages are essential for the inflammatory response in BCG treatment. Dendritic cells and T lymphocytes present among the spleen cells may be responsible for the small portion of inflammatory cytokines that continue to be produced even after depletion (Fig. 9).

### MB49 infected with BCG activates a MyD88-dependent inflammatory response in macrophages

**Figure 10:**
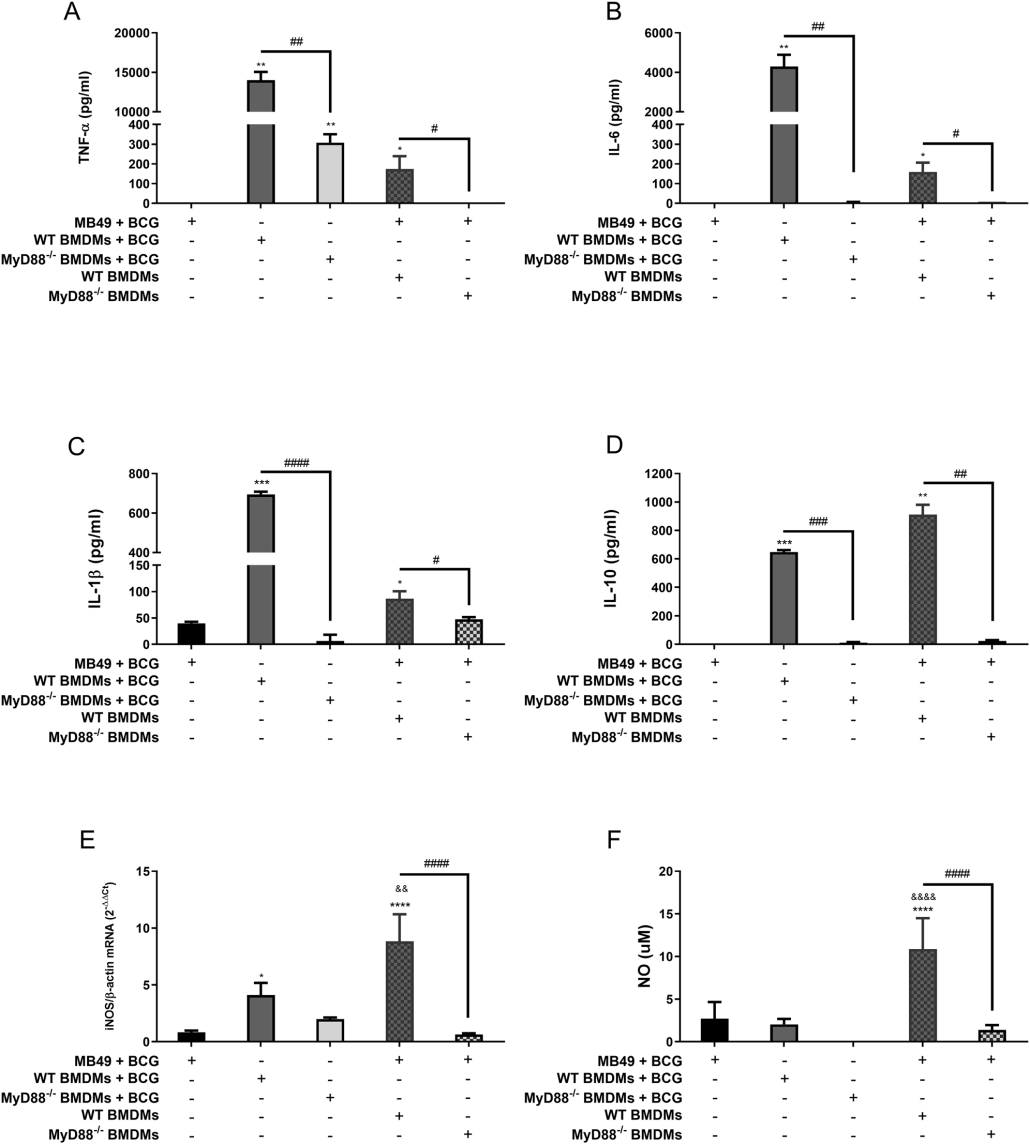
MB49 harboring BCG activates a MyD88-dependent inflammatory response in macrophages. MB49 cells used for co-culture were previously infected with BCG (MB49 + BCG) for 24 h and washed to remove free BCG in the supernatant, previously to the addition of C57BL/6 WT or MyD88^−/−^ macrophages in the co-culture. Co-cultured cells were maintained together for 24 h (**A**–**E**) or 48 h (**F**), when the samples were collected for analysis. MB49 or macrophages were also infected separated (not in co-culture) and used as controls. Measurement of TNF-α (**A**), IL-6 (**B**), IL-1β (**C**) and IL-10 (**D**) production in the supernatants after 24 h were performed by ELISA. (**E**) iNOS mRNA expression in co-cultured cells after 24 h was evaluated by qPCR. All qPCR results were relative to β-actin mRNA as a normalizer and C57BL/6 WT BMDM (mock) used as control. (**F**) NO production was evaluated by the Griess method in supernatants after 48 h of stimulation. Mock treated data are not shown in graphs. The values are representative of at least three independent experiments. *Statistically significant compared to MB49 + BCG, *P ≤ 0.05, **P ≤ 0.01, ***P ≤ 0.001, ****P ≤ 0.0001. ^#^Statistically significant data comparing C57BL/6 WT and MyD88^−/−^ BMDMs, ^#^*P* ≤ 0.05, ^##^*P* ≤ 0.01, ^###^*P* ≤ 0.001, ^####^*P* ≤ 0.0001. ^&^Statistically significant data comparing C57BL/6 WT BMDM in co-culture or not, ^&&^*P* < 0.01, ^&&&&^*P* ≤ 0.0001.

Tumor development depends on the modulation of the immune response in the TME, considering that M1 macrophages work to control tumors while M2 macrophages prevents inflammation favoring tumor growth. To confirm if BCG-infected tumor cells depend on MyD88 in the macrophages to polarize an inflammatory profile, we performed in vitro co-culture using infected MB49 with BMDMs from C57BL/6 WT or MyD88^−/−^ mice. MB49 cells used for co-culture were previously infected (BCG MOI 40) for 24 h followed by the addition of WT or MyD88^−/−^ BMDMs and maintained in co-culture for 24 or 48 h. BMDMs from WT or MyD88^−/−^ (not in co-culture) were infected at the same time as the co-culture and maintained for controls. Only WT BMDMs in co-culture with BCG-infected MB49 cells were able to produce significant levels of inflammatory mediators such as TNF-α, IL-6, IL-1β, NO and *iNOS* mRNA expression compared to BMDMs from MyD88^−/−^ mice. We also detected the production of IL-10 in WT BMDMs, probably acting to counterbalance the inflammatory response. MyD88^−/−^ BMDMs did not show a significant cytokine response in the presence of infected MB49 cells (Fig. 10A–F). MB49 and BMDMs in co-culture without BCG infection were not properly activated (data not shown). BCG depends on the MyD88 pathway in WT macrophages infected and also in WT BMDMs in co-culture with infected MB49 to activate the inflammatory status of these cells. The mechanism of cell activation in the TME by MB49 infected with BCG needs to be better understood to improve immunotherapy strategies in order to activate an effective anti-tumor response.

## Discussion

The negative correlation between tuberculosis and the incidence of cancer in patients was first related by Pearl in 1929^43^ opening new perspectives for the development of a immunotherapy using microorganisms to treat bladder cancer^44^. Different microorganisms^45^ and viruses have also been tested for cancer treatment^46,47^. Recently, herpes simplex virus type 1 (HSV-1)-based oncolytic viruses engineered to express granulocyte–macrophage colony-stimulating factor (GM-CSF) was approved to treat advanced melanoma^48^. Immunotherapy efficiency depends not only on the microorganism itself but especially on the individual's immune system. Many studies try to explain the role of the immune response in BCG immunotherapy. The new concept of "Trained Immunity" brings evidence of how the innate immune response is essential for the heterologous effect of BCG treatment against cancer^49^. BCG treatment has already been experimentally tested for other types of cancer such as leukemia, melanoma and breast cancer^50,51^. In this study, we investigate the role of important host innate immune molecules in BCG treatment for tumor reduction in a subcutaneous murine bladder cancer model (MB49).

BCG treatment works for tumors already grown (data not shown), but with a more prominent effect in early treatment right after implantation of subcutaneous tumors as showed before by Gan^35^ and confirmed by our data. The weekly treatment with BCG in C57BL/6 WT mice leads the tumors practically to disappear after 3 treatments. Several studies have shown that intratumoral BCG injection is the most effective route for cancer immunotherapy. BCG attaches via fibronectin and α5β1 integrin to the surface of the bladder cancer cells and also to benign urothelial cells^6,52–54^. The bacteria is internalized and induces different levels of surface molecules, such as MHC-II, CD1, CD80 and ICAM-1^55,56^. The recognition and processing of BCG by tumor cells are reported as essential for subsequent induction of the antitumor response. However, many tumor cell lines are deficient or lost the ability to activate important immune pathways after many passages in culture. The ability to internalize BCG and initiate an immune response varies according to each tumor cell line^57^. Initially, we proposed to investigate whether MB49 cell line was able to respond to BCG infection or agonists specific for different innate immune pathways such as Pam3CSK4 (TLR2), ultrapure LPS (TLR4), CpG-ODN (TLR9), dsDNA90 (cGAS-STING) or cGAMP (STING). MB49 cells were not able to activate any inflammatory response (TNF-α, IL-6, CXCL-10 or IFN-β) unlike macrophages, revealing an impaired response related to some TLRs receptors and also to cGAS-STING pathway. However, MB49 was activated by poly I:C via TLR3 producing large amounts of CXCL-10 and expressing higher levels of *IFN-β*. Additionally, the cGAS-STING pathway was not activated by dsDNA in MB49 cells, but responded to the direct stimulus of cGAMP suggesting that cGAS is not functional in this tumor cell line. The expression of cGAS and/or STING is recurrently suppressed by DNA hypermethylation in a variety of cancer cell lines, leading to an inability to activate the production of STING-dependent cytokines, important to suppress tumor development^37–40^. We did not detect direct MB49 activation even with high BCG doses (MOI 10, 20 and 40). MB49 cell line itself does not seem to be an effective inducer of the innate immune system in response to BCG and other agonists.

We confirmed that BCG was able to infect MB49 cells; however, the different BCG MOI used did not induce significant cell death that could justify the low levels of cytokines released. Our hypothesis was that BCG did not directly stimulate the innate immune response in MB49, but the infected tumor cells could be important to activate other phagocytic cells present in TME leading to a systemic response. After euthanized the mice, we isolated spleen cells from treated and untreated animals and co-cultured with previously infected MB49 cells (MB49 + BCG). The results reinforce the inability of MB49 to produce inflammatory cytokines in response to high BCG CFU (MOI 40). However, we detected an increased inflammatory response (TNF-α, IL-6, IFN-γ, CXCL10 and NO) in the co-culture of MB49 infected with BCG and spleen cells from treated mice. Even though the intrinsic innate immune response from MB49 cells does not seem to be important, BCG immunotherapy can activate the extrinsic innate responses from the immune cells present in TME leading to a subsequent BCG-specific systemic adaptive immunity^55,56,58^.

The effect of BCG in urothelial cells or antigen-presenting cells (APCs) depend on the recognition of bacterial PAMPs by extracellular TLRs^10,49,59^ or the mycobacterial DNA sensing by endosomal TLRs or STING^60,61^. The majority of the studies suggest the relevance of these immune pathways according to the cytokines measured in urine or peripheral blood from patients after BCG treatment^62–64^. We specifically investigated the role of different TLRs and the cGAS-STING pathway using KO mice in BCG tumor treatment using MB49 syngeneic tumor model. *Mycobacterium tuberculosis* and *M. bovis* activate a cGAS-independent STING pathway dependent on the detection of c-di-AMP (cyclic-di-adenosine monophosphate) in the cytoplasm^17^. Type I IFN production in tumors promoted by the activation of the cGAS-STING pathway^22^ in response to specific agonists^20,21,65^ and even fractionated radiotherapy^24,25,66^ has been shown to enhance the response not only in the local tumor but also recruiting lymphocytes and promoting the systemic response in the abscopal tumor. In vitro, MB49 cells and macrophages showed significantly amount of CXCL10 and *IFN-β* expression in response to cGAMP and poly:IC. Even though cancer cells containing double-stranded DNA or cyclic di-nucleotides (CDNs) could not direct activate intrinsic STING, they are able to stimulate the extrinsic STING pathway present in phagocytic cells and to promote antigen cross-presentation^21^. Knowing the importance of type I IFN in the antitumor response, we decided to investigate the effect of BCG treatment in mice deficient for cGAS, STING and TLR3, molecules involved in IFN-β production. cGAS^−/−^, STING^−/−^ and TLR3^−/−^ mice showed similar tumor reduction as C57BL/6 WT animals after three treatments with intratumoral BCG, discarding the importance of these receptors in BCG-induced tumor regression. In order to confirm if IFN is involved in BCG anti-tumor immunotherapy we performed experiments using IFNAR^−/−^ mice and observed that BCG tumor treatment is independent on IFN signaling. Recent study reported the production of a recombinant BCG expressing a STING agonist (c-di-AMP), capable of strongly activate inflammatory macrophages (M1 profile) and induce trained immunity when compared to wild type BCG^67^. The use of agonists, especially those that activates type I IFN, in concert with BCG could potentiates the activation of STING and TLR3^68,69^ pathways to improve the effect of the immunotherapy.

The majority of TLRs depends on the MyD88 adaptor molecule, except for TLR3 that recruits TRIF and TLR4 that depends on both MyD88 and TRIF. MyD88 activates MAPKs and NF-κB whereas TRIF activates IRF3 leading to type I IFN production^13^. Herein, we decided to investigate the role of four different MyD88-dependent single TLRs (TLR2^−/−^, TLR4^−/−^, TLR7^−/−^ and TLR9^−/−^) in BCG tumor treatment. All four TLRs KO mice responded similar to WT after three intratumoral BCG treatment. TLR4^−/−^, TLR7^−/−^ and TLR9^−/−^ untreated tumors presented significantly small volume compared to WT untreated tumors indicating that these TLRs could be involved in normal tumor development. The results did not show any relevance for the single TLRs tested here in BCG tumor treatment. Therefore, our strategy was to eliminate a set of TLRs related to endosomal pathway using triple deficient mice (TLR3/7/9^−/−^) or MyD88^−/−^ deficient animals. TLR3/7/9^−/−^ mice showed a delay in tumor regression and partial tumor volume reduction compared to WT mice. The lack of MyD88 molecule completely eliminated the effect of BCG in tumor volume reduction. MyD88 drives a wide role as an adapter molecule for TLRs and also interleukin-1 receptor (IL-1R) family^70^. The importance of MyD88 to the inflammasome complex activation can be related to the first signal after TLRs induction or downstream IL-1R signaling^71^. Our complementary data using IL-1R^−/−^, caspase1/11^−/−^ and Gasdermin-D^−/−^ mice demonstrated that MyD88 role in BCG treatment does not involve specifically the activation of the inflammasome complex or IL-1R. All results with different innate immune pathways suggest that MyD88 is essential for BCG-tumor treatment depending on a synergistic effect involving several MyD88-dependent TLRs working simultaneously and/or a TLR-independent effect.

After the internalization of BCG by tumor or phagocytic cells or even the phagocytosis of infected tumor cells by macrophages and dendritic cells, the antigens are presented to T cells via MHC-II^55,56^. T cells recognizes BCG-specific antigens and possibly tumor-specific antigens as well. The importance of CD4+ and CD8+ T lymphocytes in BCG treatment was demonstrated in experiments using lymphocyte-deficient animals^58,72,73^. BCG-specific responses are important to target the cell infiltrate profile in the tumor by recruiting effector cells like CD8 + cytotoxic T-cells (CTLs), macrophages, neutrophils, natural killer (NK) cells and others^7,74^. BCG immunotherapy affects cellular infiltrate in TME and also activates the inflammatory response with a polarization of type 1 macrophages (M1)^30,75^, production of inflammatory cytokines^76–79^ and nitric oxide^80^. We therefore investigated the immune cells infiltrate in WT and MyD88^−/−^ mice after two BCG treatments. BCG treatment in WT mice significantly increase the presence of macrophages, neutrophils, CD8+ T lymphocytes and NKT cells in TME. Our results confirm that intratumoral BCG has the potential to increase the inflammatory macrophage population (M1) and to diminish even more the anti-inflammatory profile (M2). We also detected upregulation of *iNOS* mRNA expression unlike arginase in tumors from WT mice. TME cell infiltrate in MyD88^−/−^ mice did not alter after BCG treatment. Additionally, we performed co-culture experiments with MB49 and macrophages-depleted spleen cells and the results show a reduction on inflammatory cytokines after depletion, reinforcing that macrophages are essential for the immune response during BCG tumor treatment. Finally, we confirm the importance of MyD88 in macrophages showing that BCG induces the release of inflammatory cytokines (TNF-α, IL-6, IL-1β) and nitric oxide by WT macrophages in co-culture with infected tumor cells, but not in MyD88^−/−^ BMDMs. Tumor-infiltrating immune cell subpopulations dictate the immunotherapy outcome^81^ and M2 profile is associated with poor prognosis of bladder cancer^28–30^. The production of nitric oxide after treatment with BCG and its cytotoxic effect was already demonstrated^80,82,83^. On the other hand, BCG also releases regulatory cytokine such as IL-10 allowing a counterbalance of the inflammatory response favoring the tumor control without so much damage caused by an exacerbated immune response^84–86^. Overall, our results suggest the essential role of MyD88 to generate an efficient BCG anti-tumor response. This research consolidates the knowledge related to the immune response induced by BCG in the bladder tumor model and may contributes to the improvement of cancer immunotherapy.

## Methods

### Animals

C57BL/6 wild-type mice were obtained from the Federal University of Minas Gerais (UFMG) animal facility. Mouse deficient for the different Toll-like receptors (TLR2^−/−^, TLR3^−/−^, TLR4^−/−^, TLR7^−/−^, TLR9^−/−^, TLR3/7/9^−/−^) and the adaptor molecule MyD88 (MyD88^−/−^) were provided by Dr. Shizuo Akira (Osaka University, Osaka, Japan), the interleukin-1 receptor (IL-1R^−/−^)^87^, the caspase-1 and -11 enzymes (Caspase1/11^−/−^)^87^, the protein gasdermin D (Gasdermin^−/−^)^88^, the interferon α/β receptor (IFNAR^−/−^), the STING receptor (STING^−/−^)^89^ and cGAS (cGAS^−/−^) were described previously and housed in a specific pathogen-free laboratory facility. STING^−/−^ and cGAS^−/−^ mice were provided by Dr. Glen N. Barber (University of Miami, Miami, USA). Female mice were used at 8–10 week of age. Euthanized mice were anesthetized with a solution containing 25% Ketamine and 9% Xylazine in 0.9% NaCl. Experiments were performed according to protocols that were approved by the Ethics Commission on Animal Use (CEUA) from the Federal University of Minas Gerais (UFMG) under permit #372/2019.

### BCG

The *Mycobacterium bovis* BCG strain Moreau and Pasteur was used in all the experiments in vitro and in vivo. BCG was grown in Middlebrook 7H9 broth that contained 0.05% Tween 80 and 0.2% glycerol and was supplemented with 10% albumin-dextrose-catalase. Cultures were harvested by centrifugation at the mid-exponential phase, suspended in saline containing 0.05% Tween 80, and stored at − 80 °C until use^90^.

### Cancer cell culture

The MB49 mouse urothelial carcinoma parental cell line was kindly provided by Dr. Thomas F. Gajewski (The University of Chicago Medicine**,** Chicago, USA). MB49 cells were grown in DMEM (Gibco) supplemented with 10% heat-inactivated FBS (Gibco), 1% HEPES, penicillin G sodium (100 U/ml) and streptomycin sulfate (100 μg/ml).

### Murine heterotopic tumor model

Subcutaneous tumor injections were carried out by inoculating mice with 5 × 10^5^ MB49 cells diluted in 100 μl of PBS on the right shaved flank. Intratumor treatment with BCG (8 × 10^6^ CFU diluted in 60 μl of PBS) or 60 µl PBS control (mock) was carried out 24 h after tumor implantation^35^ and repeated once a week for a total of two doses (euthanized 15 days pos-tumor injection) for TME analyses (tumor flow cytometry and qPCR) or three doses (euthanized 22 days pos-tumor injection) for tumor growth. Tumor development was measured weekly (8, 15 and 22 days pos-tumor injection) with a digital caliper and calculated with the formula Volume = (length × width^2^)/2^21^. Mice were euthanized if the tumor volume was greater than 800 mm^3^.

### Bone marrow-derived macrophages (BMDMs)

To generate BMDMs, bone marrow cells from mice were removed from the femurs and tibias of the animals and cultured in DMEM (Gibco) with 10% FBS, 1% HEPES, penicillin G sodium (100 U/ml), streptomycin sulfate (100 μg/ml), and 20% L929 cell conditioned medium in petri dishes (1 × 10^7^ cells). The cells were cultured at 37 °C in an atmosphere of 5% CO_2_. After 4 days, 10 ml of complete fresh medium was added. At day seven in culture, the cells had completely differentiated into macrophages. Macrophages were seeded in 6-well plates (5 × 10^5^ cells/well), 24-well plates (2 × 10^5^ cells/well) or 96-well plates (1 × 10^5^ cells/well) and used for in vitro studies as previously described^90^.

### Spleen cells culture

Cells obtained from the spleens of mice that developed subcutaneous tumor treated with BCG or mock (PBS) were washed with saline and the erythrocytes were lysed with a hemolytic solution ACK (155 mM NH_4_Cl, 10 mM KHCO_3_, pH 7.2). Spleen cells were washed with PBS, resuspended in complete DMEM medium, filtered using a cell strainer, counted and seeded at 24-well plates for the in vitro co-culture experiments as shown previously^90^.

### Co-culture in vitro experiments

MB49 cells used for co-culture were previously seeded in 24-well plates (2 × 10^5^ cells/well) and infected with BGC (MOI 40). After 24 h, the supernatant was removed and the cells were washed to remove free BCG. The same amount of BMDMs (2 × 10^5^ cells/well) or 1:2 splenocytes (4 × 10^5^ cells/well) were add together with infected or non-infected MB49 cells. The co-culture was maintained for another 24 h or 48 h at 37 °C in an atmosphere of 5% CO_2_. MB49 cells, BMDMs and splenocytes were infected with BCG (MOI 40) at the same time as we started the co-culture and cultured for 24 h or 48 h as a control.

### In vitro infection and measurement of bacterial intracellular growth

BMDMs or MB49 cells were infected with *M. bovis* BCG Moreau with the determined MOI in 400 μl/well of DMEM supplemented with 10% FBS and 1% HEPES. Cells were incubated for 6 h, 24 h or 48 h at 37 °C in a 5% CO_2_ atmosphere. To quantify the number of intracellular bacteria, cells were lysed immediately after the specified period of time with 0.1% saponin (Sigma-Aldrich). Serial dilutions were plated in Middlebrook 7H11 agar medium that was supplemented with 10% oleic acid-albumin-dextrose-catalase, and the CFUs (colony forming units) were counted after 3–4 weeks of incubation at 37 °C as previously described^90^.

### Cell activation and cytokine release measurements

MB49 cells, BMDMs or splenocytes were infected with BCG or stimulated with different agonists, such as Pam3CSK4 (1 µg/ml), ultrapure LPS (100 ng/ml), CpG-ODN (1 µg/ml), PolyI:C (3 µg/ml), dsDNA90 (3 µg/ml) and cGAMP (6 µg/ml). PolyI:C, dsDNA and cGAMP transfection was performed with Lipofectamine 2000 (3 μg/ml) following manufactures instructions. Culture supernatants were collected after 24 h of stimulation or co-culture and subsequently used for TNF-α, IL-6, CXCL10 (IP-10), IFN-γ, IL-1β and IL-10 cytokines analysis by ELISA (R&D Systems, Abingdon, UK) according to the manufacturer’s instructions.

### Nitrite measurement by Griess

The supernatants of 48 h co-cultured cells were used for nitrite measurement. The concentration of nitrite (NO_2_^−^), a stable metabolite of NO, was measured using Griess reagent (1% sulfanilamide and 0.1% naphthyl ethylenediamine dihydrochloride in 2.5% phosphoric acid). Briefly, 50 μl of cell culture supernatants were mixed with 50 μl of Griess reagent in 96-wells plate. Subsequently, the mixture was incubated protected from light at room temperature for 20 min and the absorbance was measured at 550 nm in a microplate reader. Fresh culture medium was used as blank. The quantity of nitrite was determined from a sodium nitrite (NaNO_2_) standard curve as previously shown^90^.

### Lactate dehydrogenase release assay

MB49 cells were seeded into 24-well plates (2 × 10^5^ cells/well) and infected with BCG (MOI 10, 20 and 40). After 24 h of infection, supernatants were harvested for analysis of lactate dehydrogenase (LDH) release by dying cells. The cultured cells were lysed using M-PER Mammalian Protein Extraction Reagent (Thermo Fisher Scientific). Total LDH was determined by supernatant plus protein cell lysate released LDH. Dying cells were calculated by the ratio: supernatant LDH/total LDH. LDH was quantified using the CytoTox 96 LDH-release kit (Promega, Madison, WI), according to the manufacturer’s instructions as previously demonstrated^91^.

### Quantitative real-time PCR (qPCR)

Tumor tissue were collected, minced and homogenized in TRIzol reagent (Invitrogen, Carlsbad, CA, USA) to isolate total RNA accordingly to manufacturer instructions. Alternatively, MB49 cells, BMDMs or splenocytes were stimulated in 24-well plates and homogenized in TRIzol reagent (Invitrogen). Reverse transcription of 2 μg of total RNA was performed in a final volume of 20 μl containing oligo-dT (0.5 μg/μl), dNTP 10 mM, DTT 0.1 M, buffer 5× and reverse-transcriptase (2 U per reaction), using the following cycling parameters: 42 °C for 60 min and 70 °C for 15 min. Quantitative real-time PCR was conducted in a final volume of 10 μl containing the following: SYBR Green PCR Master Mix (Thermo Fischer Scientific, Waltham, MS, USA), oligo-dT cDNA as the PCR template, and 5 μM of primers^90^. The PCR reaction was performed with QuantStudio 3 Real-Time PCR System (Thermo Fischer Scientific). Primers sequences were as follows: β-actin forward, 5′-GGC TGT ATT CCC CTC CAT CG-3′; β-actin reverse, 5′-CCA GTT GGT AAC AAT GCC ATG T-3′; 18S forward, 5′-CGT TCC ACC AAC TAA GAA CG-3′; 18S reverse, 5′-CTC AAC ACG GGA AAC CTC AC-3′; IFN-β forward, 5′-AGC TCC AAG AAA GGA CGA ACA T-3′; IFN-β reverse, 5′-GCC CTG TAG GTG AGG TTG ATC T-3′; iNOS forward, 5′-AGC ACT TTG GGT GAC CAC CAG GA-3′; iNOS reverse, 5′-AGC TAA GTA TTA GAG CGG CGG CA-3′; Arginase forward, 5′-TGA CAT CAA CAC TCC CCT GAC AAC-3′; Arginase reverse, 5′-GCC TTT TCT TCC TTC CCA GCA G-3′. Data were analyzed using the threshold cycle (ΔΔ*C*t) method and they were presented as relative expression units after normalization to the house keeping genes (β-actin or 18S) as previously demonstrated^92^. PCR measurements were conducted in triplicate.

### Evaluation of cellular infiltrate in TME by flow cytometry

Mice were euthanized 15 days after tumor cells injection. The resulting tumor was dissected, minced and incubated in 200 U/ml collagenase IV (Gibco) shaking at 1000 rpm at 37 °C for 1 h. Tumor dissociated cells were filtered using cell strainer, centrifuged at 1200 rpm for 5 min and the pellet was suspended in DMEM medium. Tumor microenvironment (TME) cells were analyzed by flow cytometry (1 × 10^6^ cells/well). Different antibodies mix (anti-mouse) were used for evaluation of myeloid cells, lymphocytes, activation status or M1/M2 markers. Briefly, cells were incubated for 20 min with anti-mouse CD16/32 to block Fc receptors (BD Bioscience, Franklin Lakes, NJ, USA) in FACS buffer (PBS, 0.25% BSA, 1 mM NaN3) and were stained for surface markers as previously shown^90^. The following conjugated antibodies were used: anti-CD11c FITC conjugated (clone HL3; BD Bioscience), anti-CD8 FITC conjugated (clone 53-6.7; BD Bioscience), anti-CD80 FITC conjugated (clone 16-10A1; BD Bioscience), anti-Ly6G PE conjugated (clone 1A8; BD Bioscience), anti-NK1.1 PE conjugated (clone PK136; BD Bioscience), anti-CD163 PE conjugated (clone TNKUPJ; Invitrogen), anti-F4/80 Biotin conjugated (clone BM8; eBioscience), Anti-CD3e Biotin conjugated (clone 500A2; BD Bioscience), anti-Ly6C APC conjugated (clone HK1.4; Invitrogen), anti-CD11b APC-Cy7 conjugated (clone M1/70; BD Bioscience), anti-CD4 APC-Cy7 conjugated (clone GK1.5; BD Bioscience) and anti-F4/80 PE conjugated (clone T45-2342; BD Biosciences). Streptavidin was added where necessary. The appropriate isotype controls were used. Attune Acoustic Focusing Cytometer (Life Technologies, Carlsbad, CA, USA) was used to collect more than 100,000 events and data were analyzed using FlowJo Software (Tree Star, Ashland, OR, USA). Gating strategy is shown in Supplementary Figs. S4 and S5.

### Macrophages depletion from spleen cells

C57BL/6 WT mice treated with standard intratumoral BCG (as shown in Fig. 1A) were euthanized for the spleen cells suspension preparation as we explained previously. A determined number of cells from a pool of spleens were submitted to a negatively select macrophages depletion protocol. Anti-PE MicroBeads (Miltenyl Biotec) were used for the indirect magnetic labeling and separation of spleen cells labeled with anti-F4/80 PE conjugated primary antibody (clone T45-2342; BD Biosciences) accordingly to manufacturer instructions. Spleen cells before and after macrophages depletion were co-cultured with MB49 for cytokines detection by ELISA after 24 h. The percentage of depletion was addressed by flow cytometry.

### Statistical analysis

Results are presented as the mean ± SD. Statistically significant differences among the results obtained were evaluated by 2-way ANOVA followed by the Bonferroni post hoc test (*P* < 0.05), 1-way ANOVA followed by the Tukey post hoc test (*P* < 0.05) or the Student *t*-test (*P* < 0.05). Statistical analysis was performed using GraphPad Prism 5.0 (GraphPad Software, San Diego, CA, USA).

### Ethical approval

I hereby confirm that all methods were carried out in accordance with relevant guidelines and regulations. This study was also carried out in compliance with the ARRIVE guidelines. All the protocols adhered to the guidelines on animal ethics/regulations.

## Supplementary Information

Supplementary Figures.

## Data Availability

All relevant data are within the manuscript and its Supplementary Material.
