# Supplementary material for: MyD88-dependent BCG immunotherapy reduces tumor and regulates tumor microenvironment in bladder cancer murine model
Source: Sci Rep. 2021 Aug 2;11:15648. doi: 10.1038/s41598-021-95157-6 (PMC8329301; doi:10.1038/s41598-021-95157-6)
Supplement: Supplementary file 1 — Supplementary Figures. [file 41598_2021_95157_MOESM1_ESM.pdf]

MyD88-dependent BCG immunotherapy reduces tumor and regulates tumor microenvironment in bladder cancer murine model

Nina M. G. P. de Queiroz[1], Fabio V. Marinho[1], Ana Carolina V. S. C. de Araujo[1], Julia S. Fahel[1], Sergio C. Oliveira[1, 2,*]

[1]Departamento de Bioquímica e Imunologia, Instituto de Ciências Biológicas, Universidade Federal de Minas Gerais, Belo Horizonte, MG, Brazil.

[2] Instituto Nacional de Ciência e Tecnologia em Doenças Tropicais (INCT-DT), CNPq MCT, 31270-901, BA, Brazil.

*Correspondence and requests for materials should be addressed to S.C.O (e-mail: scozeus1@gmail.com).

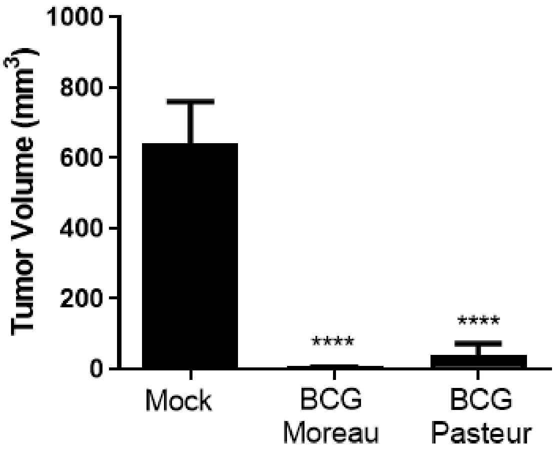

Figure S1. Subcutaneous tumors treated with BCG Moreau and Pasteur strains. Subcutaneous syngeneic tumor model MB49 ($5x10^5$ cells) injected on the right flank of C57BL/6 mice. After 24 hrs of tumor injection, mice received the first intratumoral treatment with BCG Moreau ($8x10^6$ CFU), BCG Pasteur ($8x10^6$ CFU) or PBS (Mock). The treatments were performed every 7 days (total 3 doses) and the tumor volume was evaluated once a week using a digital caliper. Figure shows the comparison of final volumes after 22 days. Data represents mean and standard deviation from the results of two independent experiments. ****Statistically significant compared to mock, $P \leq 0.0001$.

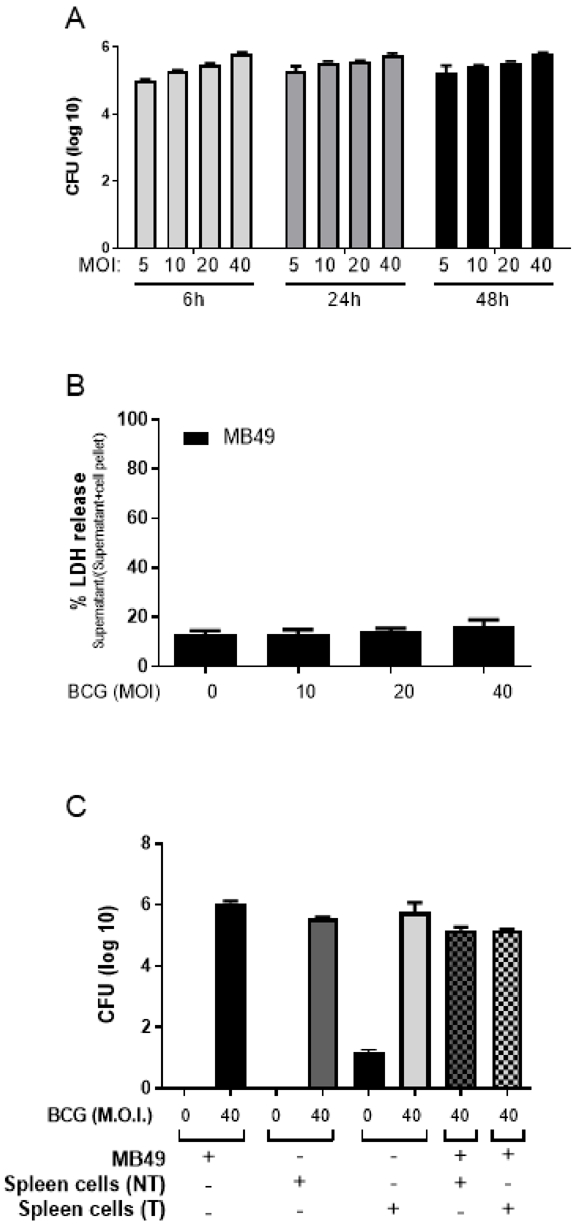

Figure S2. BCG infectivity. (A) MB49 cells were infected with different BCG MOI (5, 10, 20 or 40) during 4, 24, 48 or 72 hrs, followed by repeated washes to remove free bacteria in the medium and subsequent analysis of the intracellular bacterial by CFU. **(B)** Dying cells were measured by the ratio of LDH released in the supernatant to total LDH (supernatant + cell lysate) represented as a percentage of LDH release (% LDH), after infection with different CFU of BCG (MOI 0, 10, 20 and 40) for 24 hours. **(C)** Evaluation of intracellular BCG by CFU in MB49 and spleen cells from mice submitted to the subcutaneous tumor model and treated with BCG (T) or control PBS (not treated – NT).

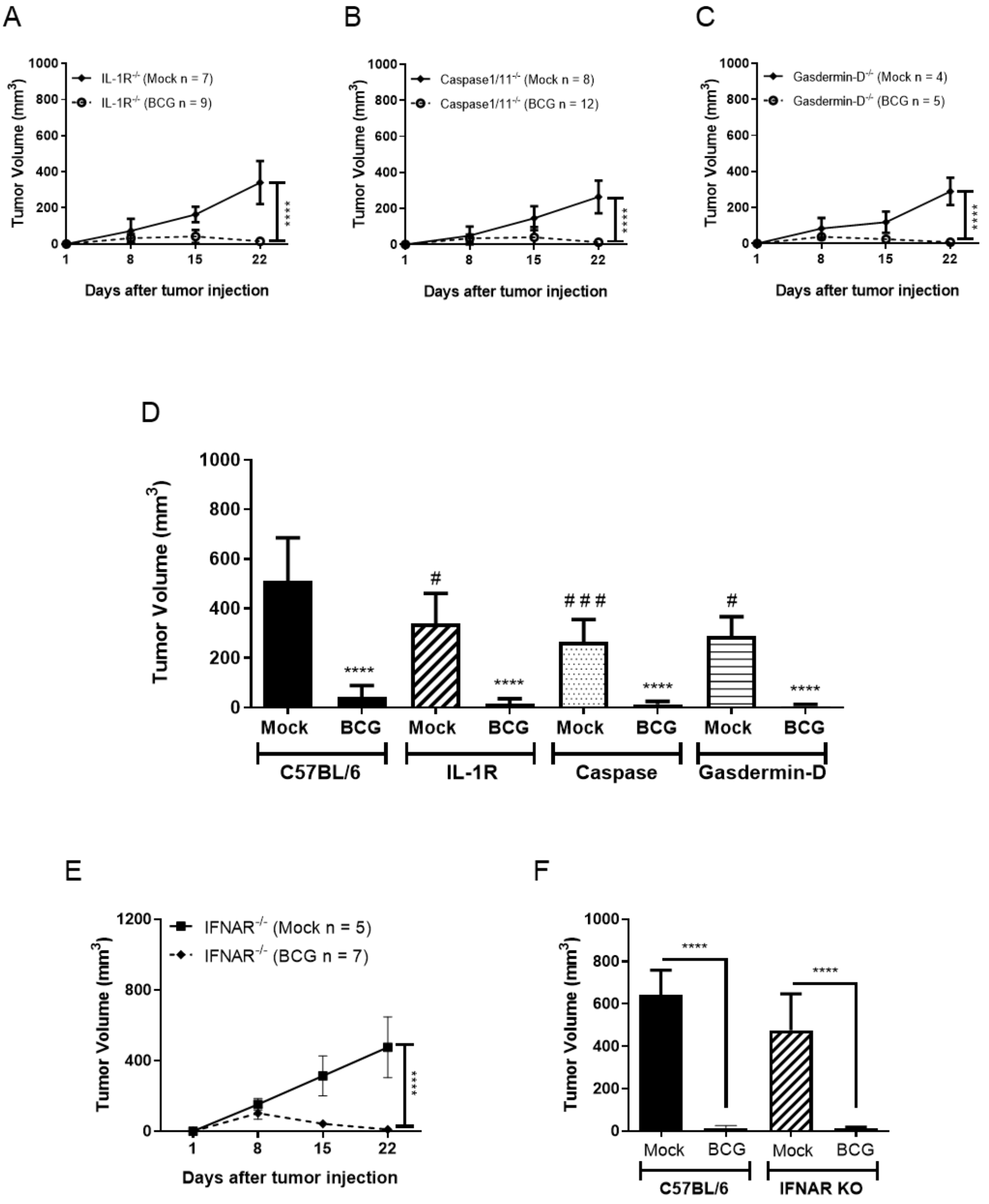

Figure S3. The role of MyD88 in the tumors treated with BCG is not related to IL-1 receptor, inflammasome pathway or IFN signaling. IL-1R$^{-/-}$, caspase1/11$^{-/-}$, Gasdermin-D$^{-/-}$ and IFNAR$^{-/-}$ mice were treated with BCG for 22 days (following the scheme in Fig. 1A). Tumor growth curves are shown for IL-1R$^{-/-}$ **(A)**, caspase1/11$^{-/-}$ **(B)**, Gasdermin-D$^{-/-}$ **(C)** and IFNAR$^{-/-}$ **(E)**. The final tumor volumes (day 22) comparing C57BL/6 WT and deficient mice are shown in Figure **D** and **F**. Data represents mean and standard deviation from the results of at least two independent experiments. C57BL/6 WT data in figure **D** represents the summary of control animals from all *in vivo* experiments comparing WT and KO mice. * Statistically significant compared to the respective untreated control, **** $P \leq 0.0001$. # Statistically significant compared to mock treated C57BL/6 WT, ### $P \leq 0.001$, #### $P \leq 0.0001$.

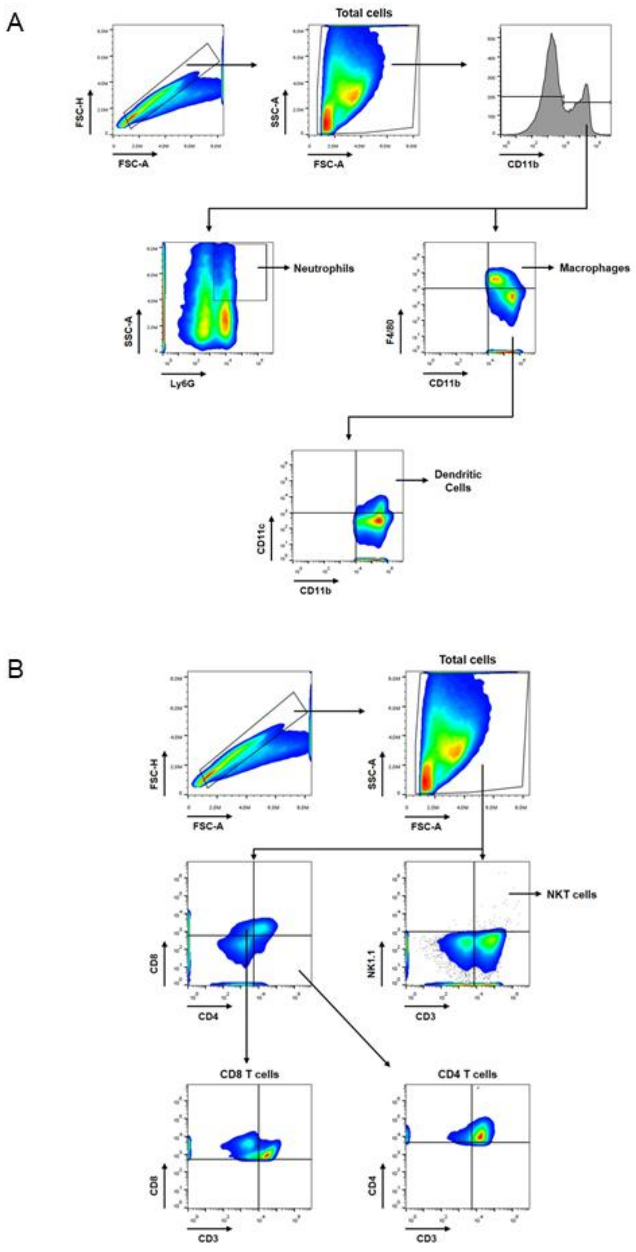

Figure S4. Gating strategy for *ex vivo* flow cytometry. (A) Tumor cells were gated for total cell population and then analyzed for expression of CD11b. The CD11b^{+} cells were then gated for Ly6G^{+}SSC^{hi} (Neutrophils) or CD11b^{+}F4/80^{+} (Macrophages). Following, the F4/80 negative cells were gated as CD11b^{+}CD11c^{+} (Dendritic cells). **(B)** Total cell population in tumors were gated for NK1.1^{+}CD3^{+} (NKT cells) or analyzed concerning CD8 or CD4 expression. The CD8^{+}CD4^{-} cells were further gated for CD3^{+}CD8^{+} (CD8^{+} T cells) and the CD8^{-} CD4^{+} cells were further gated for CD3^{+}CD4^{+} (CD4^{+} T cells).

A

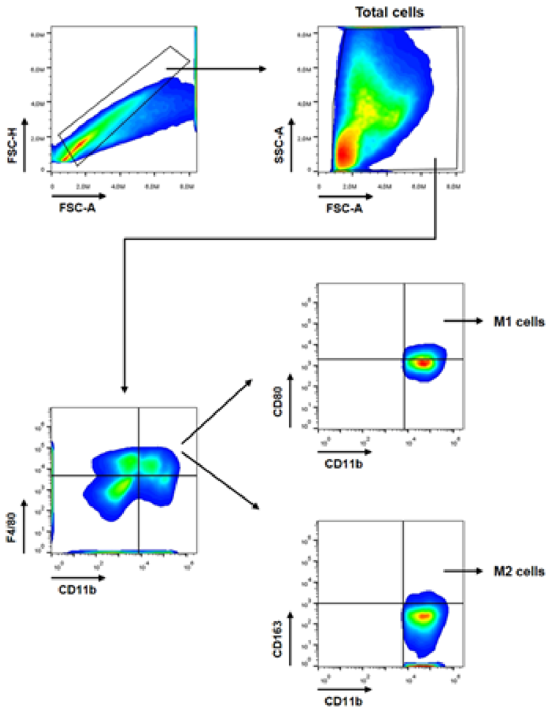

Figure S5. Gating strategy for *ex vivo* M1-M2 flow cytometry. (A) Tumor cells were gated for total cell population and the $CD11b^{+}F4/80^{+}$ cells (Macrophages) were selected. The Macrophages were further gated for $CD11b^{+}CD80^{+}$ (M1 cells) or $CD11b^{+}CD163^{+}$ (M2 cells).
